# Health and Mental Health Disparities Between National Identity Groups in Wales

**DOI:** 10.1007/s40615-020-00951-z

**Published:** 2021-01-19

**Authors:** Christopher W. N. Saville

**Affiliations:** North Wales Clinical Psychology Programme, School of Psychology, Bangor University, Brigantia Building, Penrallt Road, Bangor, Gwynedd, Wales LL57 2AS UK

**Keywords:** Wales, National identity, Mental health, Health inequalities, Ethnicity

## Abstract

Despite close links with ethnic identity and other health-relevant identities, there is surprisingly little work on national identity in the context of population health. National identity is particularly important in multi-national states, where national identity is contested and where different nationalities often reflect both distinct ethnic groups and competing civic visions of national boundaries. The present study examines health disparities between national identity groups in Wales, a constituent nation of the UK. Using data from the National Survey for Wales (*n* = 23,303), latent class analysis was used to identify national identity groups in Wales. Generalised linear mixed-effects models were then fitted to the data to identity disparities between groups in terms of self-reported general and mental health, both unconditionally and conditionally on several socio-demographic and geographic variables. Analyses identified five groups: Anglophone Welsh, British, Cymry Cymraeg (Welsh-speaking Welsh), English and Ethnically Diverse. Striking health disparities were found, with the Cymry Cymraeg and Ethnically Diverse groups reporting better health than the other groups, especially the Anglophone Welsh and the English. These disparities could not be accounted for by differences in demographic, socio-economic or geographic factors.

**Supplementary Information:**

The online version contains supplementary material available at 10.1007/s40615-020-00951-z.

## Introduction

Despite being a key component of how identity is constructed in modernity [1], national identity is a contested construct. A distinction can be drawn between ethnic and civic ideas of national identity (e.g. Zimmer [2]). Ethnic, or organic, conceptions are based upon a shared cultural inheritance: language, social practices and, in some manifestations, ancestry. Here, national identities are not something that can be chosen, and are instead determined. Civic or creedal understandings of national identity emphasise instead a voluntary commitment to a nation, often in its sense as a community of shared values [1]—identity is something that can and should be chosen. In practice however, this distinction is often more ambiguous. Yack [3] juxtaposes the apparently civic Canadian and ethnic Québécois identities, before exploring how a Québécois identity must ultimately be chosen above an equally plausible Canadian identity and how a Canadian identity is contingent on historical connections with colonial Britain and France.

Similarly, in Wales, a constituent nation of the UK, all citizens have at least two plausible national identities: Welsh and British. Furthermore, according to the 2011 UK Census, 20.8% of residents of Wales were born in England and a further 5.5% were born outside the UK [4], suggesting other possible identities. In practice, 65.9% of residents of Wales identify as Welsh, with 57.5% identifying as Welsh-only. Significant minorities, however, identify as British, English or a variety of other nationalities. Furthermore, a wide range of hybrid identities also exist, the most common of which is Welsh and British (7.1% of the population).

As with most national identities, national identity in Wales has both ethnic and civic conceptions. Historically, Welsh identity, particularly in the context of Welsh-speaking communities, was framed by figures as celebrated as John Stuart Mill as backwards and ethnic, while an Anglophone British identity was framed as forward-looking and civic [5]. However, both identities had clear ethnic components: Welsh identity being associated with the Welsh language and Methodist chapels, and British identity being associated with the English language and the Anglican Church [6]. Following a narrow vote in 1999 in favour of devolving some political powers to Wales, Brooks argues that Welsh politicians have sought to strengthen an explicitly civic and inclusive Welsh identity but, in doing so, have implicitly problematised some of the political demands of Welsh language communities as ethnic and exclusive [7]. To further complicate this issue, there is evidence that some incomers to Welsh-speaking Wales see learning Welsh as a civic act, instead of the usual view of language as an ethnic characteristic [8].

Research into Welsh identity reveals that Welsh identity is not monolithic. This research has a striking focus on place, with Welsh identity being constructed differently across the country [9]. One of the most influential models of these Welsh identities is Balsom’s Three-Wales Model [10], developed in political science. The model divides Wales into three: Welsh Wales, *Y Fro Gymraeg* (‘The Welsh-speaking country’) and British Wales. Welsh Wales covers post-industrial South Wales, particularly the coalfield that was crucial to the area’s rapid development during the industrial revolution. This identity is strongly working class, generally Anglophone, and associated with voting for the Labour Party. Y Fro Gymraeg covers much of the rural west of Wales, where the Welsh language is a living community language. Here, Welsh identity is tied to speaking Welsh and, in political terms, to support for Plaid Cymru, the Welsh nationalist party who, in the 2016 Welsh Assembly elections, won all five constituencies in Y Fro Gymraeg but only one outside it. British Wales covers the remainder of the country and, along with support for the Conservative and Liberal Democrat parties, is associated with a less confident Welsh identity [11] relative to the previous two areas, which have competing claims to heartland status—Wales’ ‘two truths’ as Raymond Williams put it [12]. Thus, in Wales, competing forms of Welshness sit alongside each other, as well as British, English and other identities.

The Three-Wales Model itself, although an enduringly useful shorthand for the various Welsh identities, has a number of limitations. Firstly, it is a model of places and not people. But, for example, Welsh speakers exist outside of Y Fro Gymraeg and the model is not clear on what to make of them. Furthermore, as Scully and Jones [13] point out, the majority of British identifiers live outside of ‘British Wales’ and only a minority in British Wales identify as British. Secondly, in proposing these regionally dominant identities, the model does not concern itself with groups that are not large enough to be a local majority. What to make of the ~ 15% of Welsh residents who do not express Welsh or British identities?

Welsh identity has been studied in its own right [9, 11] and from the perspective of electoral politics [10, 13], but, to date, not from the perspective of public health. However, there is ample reason to expect health disparities as a function of national identity in Wales. Firstly, the various Welsh identities proposed in the Three-Wales Model are highly classed, with working classness integral to Welsh Wales identity [11], while it has a more complex relationship with Welsh-speaking Welsh identities [14]. Social gradients are the rule rather than the exception in health [15], so those holding such classed identities may be at greater risk of poor health. Secondly, and relatedly, the various Welsh identities are strongly associated with particular geographical areas. Welsh Wales is strongly tied up with South Wales’ heritage of heavy industry, particularly coal mining, and poorer health in former coalfields has also been documented elsewhere [16, 17]. Y Fro Gymraeg, conversely, is predominantly rural. Rurality has been associated with better mental health in the UK [18] but may also lead to adverse consequences due to poorer access to healthcare [19]. Large geographical disparities also exist in ecological social capital, the presence of and access to resources embedded in social networks in a given locality, and these have also been associated with health outcomes [20, 21]. Thirdly, as described above, even explicitly civic national identities are often related to cultural, linguistic and other ethnic characteristics and such characteristics are often related to population health [22–24]. Fourthly, voluntary civic-type identities can also be related to health status [25].

The present paper will compare the self-rated general health and mental health of different national identity groups in Wales using a two-stage analysis of nationwide survey data. Data on national identities, Welsh language ability, ethnicity and area of residence will be clustered to identify a set of identity groups. These groups will then be compared in terms of general and mental health with and without adjusting for a number of demographic and geographical risk factors.

## Methods

### Ethics and Data Access

Ethical permission was obtained from the Bangor University School of Psychology Ethics Committee on September 14, 2020.

### Data

The present study used the 2017–2018 [26] and 2018–2019 [27] waves of the National Survey for Wales (NSfW; *N* = 11,381 and 11,922, respectively). The NSfW is a cross-sectional face-to-face survey looking at a variety of topics, including healthcare use, arts participation, diet, alcohol use and knowledge of devolution; run by the Welsh Government. Potential respondents are sampled from randomly chosen households using postal address files, aiming to be representative of 16+-year olds living in residential households in Wales. Response rates for 2017–2018 and 2018–2019 were 55% and 54%, respectively.

Survey data were linked to area measures of poverty and population density at the lower super output area (LSOA) level, using respondent LSOA codes provided by the Welsh Government under a data access agreement. Quintile of the Welsh Index of Multiple Deprivation (WIMD; Welsh Government [28]) was used as a measure of poverty, and population density was obtained from the 2011 UK Census [4].

### Measures

The variables used to identify the different identity groups were as follows: frequency of speaking Welsh (responses: daily, weekly, less often, never and cannot speak Welsh). This variable was created by combining an item asking about ability and a second item on frequency of use (asked of those who reported being able to speak Welsh), local authority (county) of residence, whether respondents identified as Welsh, whether respondents identified as British, whether respondents identified as English (the last three items were in response to a question where respondents could select as many identities as they wished, so were non-exclusive) and self-reported ethnicity (using an adaptation of the five group system used in the 2011 UK Census: White Welsh/English/Scottish/Northern Irish/British, White other, Mixed, Asian, Black and Other; https://www.ethnicity-facts-figures.service.gov.uk/style-guide/ethnic-groups). White other and White Welsh/English/Scottish/Northern Irish/British were separated as they seemed likely to differ in terms of national identity.

For the analyses of health outcomes, the following variables were used: gender, age (grouped into seven bins: 16–24, 25–34, 35–44, 45–54, 55–64, 65–74, 75+), education (higher degree/postgraduate qualifications, first degree, A/AS levels, diplomas, etc.; O level/GCSE grades A–C, etc.; O level/GCSE grades D–G; other qualifications; trade apprenticeships; foreign qualifications; no qualifications), self-reported income (less than £10,400 a year, £10,400 to £20,799 a year, £20,800 to £31,099 a year, £31,100 to £41,499 a year, £41,500 or more a year), material deprivation (a binary measure of whether a respondent is materially deprived, based on their responses to a number of questions about whether or not they can afford various items; further details available from https://gov.wales/sites/default/files/statistics-and-research/2019-02/national-survey-wales-2017-18-poverty-deprivation.pdf), WIMD quintile of their LSOA of residence, population density of their LSOA of residence, a question measuring perceived financial pressure (“Which one of the statements on this card best describes how well you [and your family/and your partner] are keeping up with your bills and credit commitments at the moment?”, with the following response options: “Keeping up with all bills and commitments without any difficulties”, “Keeping up with all bills and commitments but it is a struggle from time to time”, “Keeping up with all bills and commitments but it is a constant struggle”, “Falling behind with some bills or credit commitments”, “Having real financial problems and have fallen behind with many bills or credit commitments” and “Have no bills”) and which region of the Three-Wales Model. Respondents lived in Y Fro Gymraeg (Ynys Môn, Gwynedd, Ceredigion and Carmarthenshire), Welsh Wales (Swansea, Neath Port Talbot, Rhondda Cynon Taf, Merthyr Tydfil, Caerphilly, Blaenau Gwent, Torfaen) and British Wales (Conwy, Denbighshire, Flintshire, Pembrokeshire, Wrexham, Powys, Monmouthshire, Newport, Vale of Glamorgan, Bridgend and Cardiff). Note that Balsom’s original delineation was on the basis of UK Parliamentary constituencies in the 1980s; thus, drawing the boundaries on the basis of local authorities may lead to some small differences, but these are very minor.

Two domains of health were analysed. First, self-reported general health was measured using the item “How is your health in general; is it…”, with the following response options: “Very good”, “Good”, “Fair”, “Bad” and “Very bad”. Following previous studies [29–31], the two ‘good’ responses were coded as zero and the other three categories were coded as one, so models estimated the risk of ‘not good’ health. Second, mental health was measured using the item “Do you have any physical or mental health conditions or illnesses lasting or expected to last for 12 months or more?”. Respondents whose responses were coded under the category “mental disorders” were scored as 1, and all other were scored 0.

### Analyses

#### Latent Class Analysis

There were two stages of analysis. Firstly, latent class analysis was used to divide respondents into the appropriate number of identity groups; secondly, the self-reported general health and mental health of these groups were compared, both crudely and after adjusting for a number of different factors, detailed below. All analyses were run in R [32].

Before latent class analysis, missing data were imputed using the Amelia package [33] for R. All variables to be used in the health analyses below, as well as the three national identity variables (ethnicity, frequency of speaking Welsh and local authority), were included as predictors. A single version of the imputed data was then used for the latent class analysis. A full multiple imputation is carried out before the health analyses, as to incorporate the output of the latent class analysis.

Latent class analysis was run using the *PoLCA* function in the R package of the same name [34]. Solutions including between one and nine groups were fitted to the data. Each solution was allowed up to 100,000 iterations and was fitted with twenty different start points to avoid local minima.

Model selection can be difficult in latent class analysis. Simulation studies recommend Bayesian information criteria (BIC), and bootstrapped likelihood tests are the best approach to determine the best fitting model [35, 36]. However, these studies had sample sizes of only 1000 in the largest simulated samples, which is an order of magnitude lower than used here. Even assuming the same underlying structure, larger sample sizes will lead to a greater number of classes [37]. Thus here, BIC was plotted in the style of a scree plot, to identify the point of inflection, after which adding additional classes does not lead to substantial changes in BIC. However, models after this point will also be examined to see how they differ, to ensure that the model space is well understood.

#### Analyses of Health

Prior to the health analyses, missing data were multiply imputed using the Amelia package [33] for R. This step was carried out after latent class analysis, ensuring that the latent class was present in the imputation model to avoid bias. Five imputations were made of missing data, using gender, education, material deprivation, dichotomised general health, mental health, ethnicity, Three-Wales Region, financial stress and the latent class variable as nominal variables; income band, WIMD quintile and age group as ordinal variables; and sampling weight and population density as numeric variables.

The health of the resulting groups was compared using a series of linear mixed-effects models. The strategy was to fit a series of models with increasing levels of adjustment for health-relevant individual and area-level factors, in order to describe the health disparities as they appear in the population but also to explore the extent to which any disparities can be explained by age, class and geography. The goal here is not to infer causal relationships between these factors and health, but to compare groups unconditionally and conditionally on some obvious health-relevant factors.

For each dependent variable, general health and mental health, seven binomial log-linked mixed-effects models were fitted. Model 1 included only the latent class analysis derived group, plus a random intercept of LSOA nested within local authority. Model 2 was as model 1 but added gender and age group. Model 3 was as model 2 but added education and income, and model 4 was as model 3 but added area-level poverty. Model 5 was as model 4 but added area-level population density (z-scored). Model 6 was as model 5 but added the Three-Wales Model region of respondents. Model 7 was as model 6 but added perceived financial pressure and material deprivation, added last as these represented respondents’ interpretations of their financial situation and so were more vulnerable to reverse causation issues with mental health. In all models, residuals were weighted by the provided sampling weights. Collinearity was assessed using the check_collinearity function of the performance package [38] for R.

In order to account for the multiple imputation, five iterations of each model were run, one for each imputation, and the point estimates and standard errors were pooled using Rubin’s rule [39], as implemented in the mi.meld function of the Amelia package.

## Results

### Missing Data

From a total 23,303 respondents to the 2017–2018 and 2018–2019 surveys, 16,764 respondents had all variables available. Seven respondents lacked data on gender, 216 lacked education data, 6195 lacked data on income (note that the question on income was introduced partway through the 2017–2018 fieldwork period. Data were missing for 3051 respondents interviewed during the period when the question was asked), 192 were missing data on material deprivation, 280 lacked data on financial pressure, 24 lacked ethnicity data, eight lacked data on Welsh language, 59 lacked data on general health and 162 lacked data on mental health. Missing data were imputed as described above.

### Latent Class Analysis

**Fig. 1:**
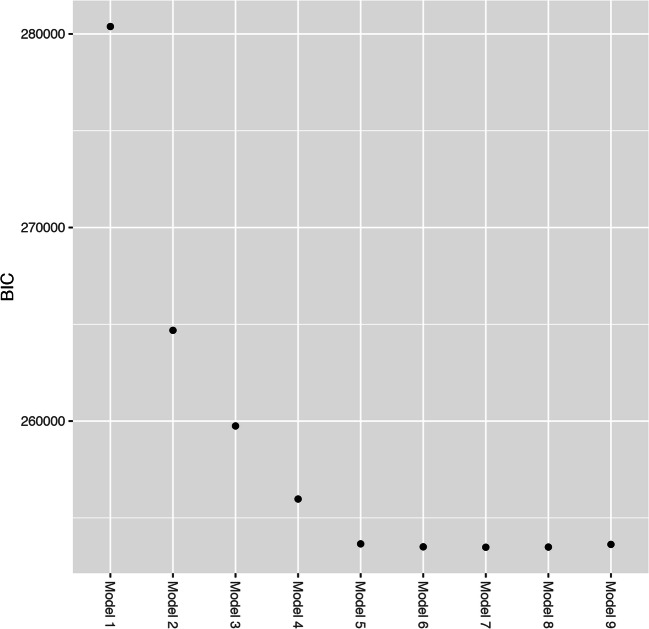
Bayesian information criteria for the nine latent class analyses. BICs decline steeply from models 1 to 5 and then plateau, although the lowest score is for model 7

Figure 1 shows a scree plot of BIC for the nine fitted models. BIC is lowest for a seven-class solution, but after model 5, BIC plateaus markedly. Thus, the main analyses reported will be for the five-class model.

**Fig. 2:**
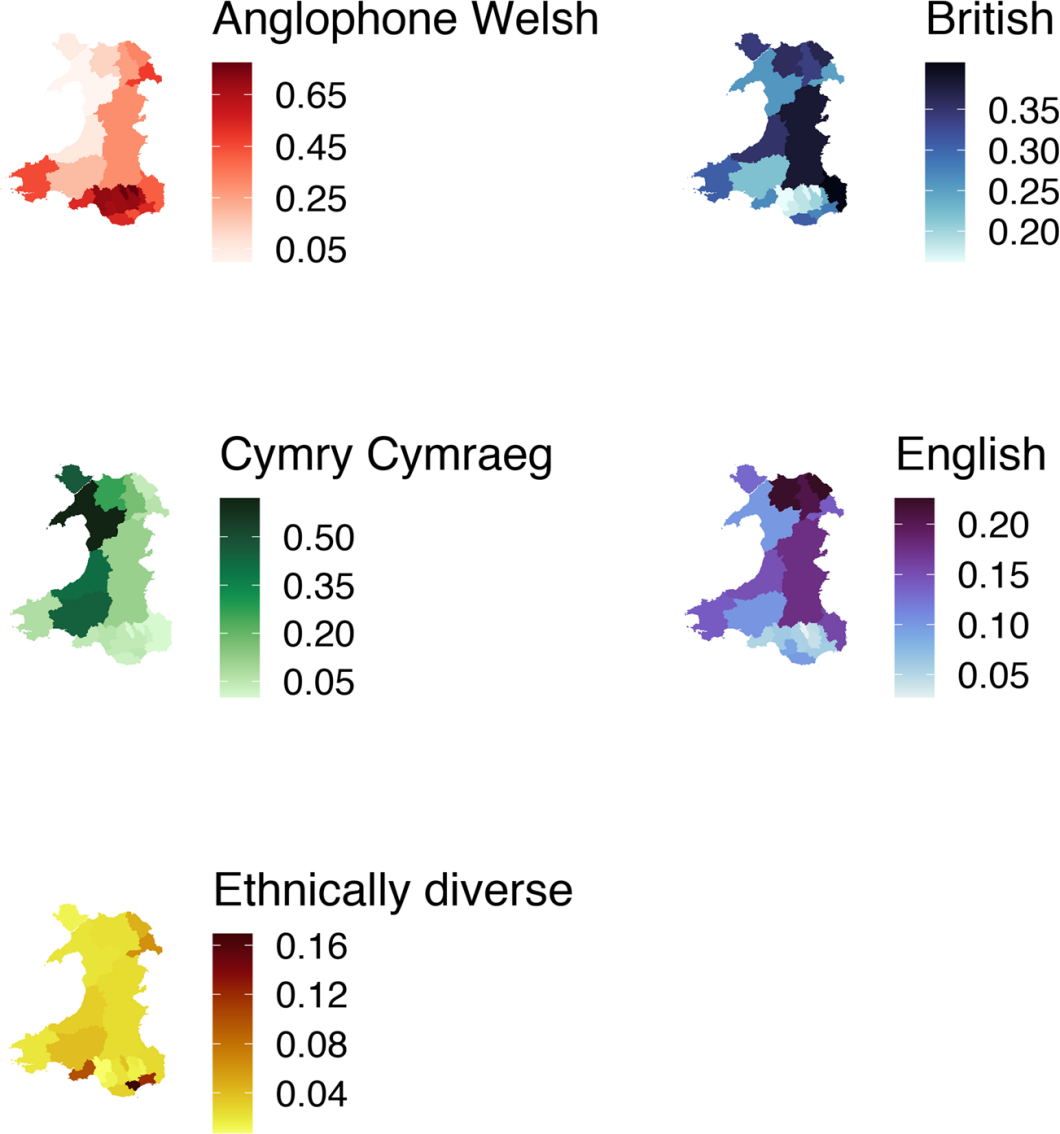
Proportions of each local authority that each identity group makes up

The five-class model is summarised in Table 1. In descending order of share of population share, they are as follows: Anglophone Welsh, British, Cymry Cymraeg (Welsh-speaking Welsh), English and Ethnically Diverse. See Fig. 2 for the distribution of each group between local authorities.

**Table 1:** Composition of each identity group in terms of variables used in latent class analysis and in analyses of health

	Anglophone Welsh (*n* = 10,425)	British (*n* = 6431)	Cymry Cymraeg (*n* = 2848)	English (*n* = 2462)	Ethnically Diverse (*n* = 1137)
Not good general health (%)	3848 (37.0)	1948 (30.3)	778 (27.4)	938 (38.2)	246 (21.7)
Mental health problems (%)	1097 (10.6)	611 (9.6)	178 (6.3)	207 (8.5)	69 (6.2)
Welsh identifying (%)	10,414 (99.9)	35 (0.5)	2830 (99.4)	73 (3.0)	17 (1.5)
English identifying (%)	38 (0.4)	419 (6.5)	25 (0.9)	2224 (90.3)	18 (1.6)
British identifying (%)	1457 (14.0)	6431 (100.0)	231 (8.1)	0 (0.0)	288 (25.3)
Ethnicity (%)					
Asian	31 (0.3)	60 (0.9)	1 (0.0)	2 (0.1)	288 (25.4)
Black	0 (0.0)	22 (0.3)	0 (0.0)	0 (0.0)	96 (8.5)
Mixed	34 (0.3)	47 (0.7)	6 (0.2)	5 (0.2)	14 (1.2)
White Welsh/English/Scottish/Northern Irish/British	10,336 (99.2)	6293 (98.0)	2828 (99.4)	2440 (99.3)	61 (5.4)
White other	21 (0.2)	0 (0.0)	9 (0.3)	10 (0.4)	528 (46.6)
Other	0 (0.0)	0 (0.0)	1 (0.0)	0 (0.0)	146 (12.9)
Frequency of speaking Welsh (%)					
Daily	61 (0.6)	289 (4.5)	2277 (80.0)	43 (1.7)	12 (1.1)
Weekly	455 (4.4)	271 (4.2)	298 (10.5)	62 (2.5)	12 (1.1)
Less often	1179 (11.3)	564 (8.8)	235 (8.3)	131 (5.3)	56 (4.9)
Never	692 (6.6)	319 (5.0)	15 (0.5)	85 (3.5)	20 (1.8)
Non-speaker	8038 (77.1)	4988 (77.6)	23 (0.8)	2141 (87.0)	1037 (91.2)
Male (%)	4778 (45.9)	2798 (43.5)	1193 (41.9)	1135 (46.1)	518 (45.6)
Age group (%)					
16–24	646 (6.2)	332 (5.2)	203 (7.1)	125 (5.1)	121 (10.6)
25–34	1189 (11.4)	717 (11.1)	333 (11.7)	197 (8.0)	285 (25.1)
35–44	1225 (11.8)	855 (13.3)	376 (13.2)	215 (8.7)	320 (28.1)
45–54	1615 (15.5)	1099 (17.1)	432 (15.2)	334 (13.6)	154 (13.5)
55–64	1931 (18.5)	1265 (19.7)	505 (17.7)	459 (18.6)	105 (9.2)
65–74	2108 (20.2)	1308 (20.3)	535 (18.8)	591 (24.0)	95 (8.4)
75+	1711 (16.4)	855 (13.3)	464 (16.3)	541 (22.0)	57 (5.0)
Education (%)					
Higher degree/postgraduate qualifications	579 (5.6)	785 (12.3)	320 (11.3)	190 (7.8)	221 (20.9)
First degree	1235 (11.9)	1287 (20.1)	499 (17.6)	309 (12.7)	243 (23.0)
A/AS levels	780 (7.5)	586 (9.2)	232 (8.2)	197 (8.1)	91 (8.6)
Diplomas, etc.	1180 (11.4)	883 (13.8)	379 (13.3)	293 (12.1)	83 (7.9)
O level/GCSE grades A–C, etc.	2050 (19.8)	1025 (16.0)	486 (17.1)	403 (16.6)	79 (7.5)
O level/GCSE grades D–G	385 (3.7)	197 (3.1)	72 (2.5)	86 (3.5)	14 (1.3)
Other qualifications	950 (9.2)	468 (7.3)	174 (6.1)	216 (8.9)	66 (6.3)
Trade apprenticeships	595 (5.7)	267 (4.2)	128 (4.5)	119 (4.9)	19 (1.8)
Foreign qualifications	2 (0.0)	8 (0.1)	1 (0.0)	5 (0.2)	84 (8.0)
No qualifications	2620 (25.3)	887 (13.9)	548 (19.3)	606 (25.0)	155 (14.7)
Income band (%)					
Less than £10,400 a year	2424 (32.3)	1332 (27.2)	587 (27.8)	630 (34.9)	266 (33.6)
£10,400 to £20,799 a year	2736 (36.5)	1744 (35.5)	736 (34.9)	637 (35.3)	280 (35.4)
£20,800 to £31,099 a year	1307 (17.4)	962 (19.6)	427 (20.2)	278 (15.4)	108 (13.7)
£31,100 to £41,499 a year	685 (9.1)	519 (10.6)	243 (11.5)	152 (8.4)	64 (8.1)
£41,500 or more a year	344 (4.6)	349 (7.1)	117 (5.5)	108 (6.0)	73 (9.2)
WIMD quintile (%)					
Most deprived 20%	2359 (22.6)	822 (12.8)	216 (7.6)	347 (14.1)	335 (29.5)
Quintile 2	2273 (21.8)	1034 (16.1)	425 (14.9)	424 (17.2)	203 (17.9)
Quintile 3	1909 (18.3)	1374 (21.4)	972 (34.1)	507 (20.6)	209 (18.4)
Quintile 4	1861 (17.9)	1741 (27.1)	810 (28.4)	731 (29.7)	192 (16.9)
Least deprived 20%	2023 (19.4)	1460 (22.7)	425 (14.9)	453 (18.4)	198 (17.4)
Population density quintile (%, people/km^2^)					
0.0–1.1	1217 (11.7)	1671 (26.0)	1128 (39.6)	720 (29.2)	82 (7.2)
1.1–5.5	2007 (19.3)	1210 (18.8)	702 (24.6)	508 (20.6)	136 (12.0)
5.5–16.5	2394 (23.0)	1168 (18.2)	461 (16.2)	424 (17.2)	173 (15.2)
16.5–36.5	2430 (23.3)	1196 (18.6)	337 (11.8)	438 (17.8)	243 (21.4)
36.5–216	2377 (22.8)	1186 (18.4)	220 (7.7)	372 (15.1)	503 (44.2)
Material deprivation (%)	1657 (16.0)	940 (14.8)	340 (12.0)	367 (15.0)	248 (22.0)
Financial strain (%)					
Keeping up with all bills and commitments without any difficulties	7120 (69.2)	4517 (70.8)	1917 (68.3)	1712 (70.3)	731 (66.2)
Keeping up with all bills and commitments, but it is a struggle from time to time	2243 (21.8)	1284 (20.1)	622 (22.2)	490 (20.1)	257 (23.3)
Keeping up with all bills and commitments, but it is a constant struggle	594 (5.8)	373 (5.8)	168 (6.0)	159 (6.5)	68 (6.2)
Falling behind with some bills or credit commitments	172 (1.7)	103 (1.6)	32 (1.1)	35 (1.4)	22 (2.0)
Having real financial problems and have fallen behind with many bills or credit commitments	99 (1.0)	53 (0.8)	20 (0.7)	20 (0.8)	16 (1.4)
Have no bills	68 (0.7)	50 (0.8)	47 (1.7)	21 (0.9)	10 (0.9)
Three-Wales Model region (%)					
British Wales	5058 (48.5)	3941 (61.3)	937 (32.9)	1683 (68.4)	772 (67.9)
Welsh Wales	5068 (48.6)	1554 (24.2)	272 (9.6)	387 (15.7)	269 (23.7)
Y Fro Gymraeg	299 (2.9)	936 (14.6)	1639 (57.5)	392 (15.9)	96 (8.4)
Local authority (%)					
Blaenau Gwent	607 (5.8)	127 (2.0)	0 (0.0)	36 (1.5)	14 (1.2)
Bridgend	604 (5.8)	158 (2.5)	36 (1.3)	81 (3.3)	12 (1.1)
Caerphilly	889 (8.5)	255 (4.0)	52 (1.8)	54 (2.2)	19 (1.7)
Cardiff	986 (9.5)	610 (9.5)	121 (4.2)	132 (5.4)	377 (33.2)
Carmarthenshire	236 (2.3)	276 (4.3)	565 (19.8)	129 (5.2)	50 (4.4)
Ceredigion	35 (0.3)	236 (3.7)	279 (9.8)	100 (4.1)	21 (1.8)
Conwy	101 (1.0)	284 (4.4)	213 (7.5)	176 (7.1)	18 (1.6)
Denbighshire	175 (1.7)	226 (3.5)	114 (4.0)	137 (5.6)	15 (1.3)
Flintshire	310 (3.0)	355 (5.5)	37 (1.3)	218 (8.9)	45 (4.0)
Gwynedd	0 (0.0)	211 (3.3)	504 (17.7)	81 (3.3)	17 (1.5)
Isle of Anglesey	28 (0.3)	213 (3.3)	291 (10.2)	82 (3.3)	8 (0.7)
Merthyr Tydfil	453 (4.3)	100 (1.6)	0 (0.0)	16 (0.6)	22 (1.9)
Monmouthshire	343 (3.3)	341 (5.3)	0 (0.0)	131 (5.3)	22 (1.9)
Neath Port Talbot	677 (6.5)	156 (2.4)	58 (2.0)	59 (2.4)	7 (0.6)
Newport	576 (5.5)	305 (4.7)	1 (0.0)	64 (2.6)	128 (11.3)
Pembrokeshire	384 (3.7)	261 (4.1)	65 (2.3)	119 (4.8)	17 (1.5)
Powys	730 (7.0)	937 (14.6)	281 (9.9)	428 (17.4)	61 (5.4)
Rhondda Cynon Taf	1037 (9.9)	279 (4.3)	65 (2.3)	83 (3.4)	32 (2.8)
Swansea	884 (8.5)	448 (7.0)	78 (2.7)	89 (3.6)	165 (14.5)
Torfaen	521 (5.0)	189 (2.9)	19 (0.7)	50 (2.0)	10 (0.9)
Vale of Glamorgan	457 (4.4)	262 (4.1)	19 (0.7)	83 (3.4)	26 (2.3)
Wrexham	392 (3.8)	202 (3.1)	50 (1.8)	114 (4.6)	51 (4.5)

All values are unweighted

#### Class 1: Anglophone Welsh

This was the largest group at 44% of the sample. They identified as Welsh, with a minority also identified as British. Although ~ 23% reported being able to speak Welsh, most of these reported speaking it infrequently. This group was concentrated in the more urban south but was widespread outside of the northwest (see Fig. 2).

#### Class 2: British

This was the second largest group at 28% of the sample. They identified as British, with small minorities also identifying as Welsh or English. They were the second most likely group to speak Welsh but were still largely monoglot Anglophone. They were concentrated in the counties of Powys and Monmouthshire and rare in the South Wales Valleys.

#### Class 3: Cymry Cymraeg

This was the third largest group at 12% of the sample. They generally identified as Welsh, and not British or English. Most (73%) spoke Welsh daily. They were concentrated in ‘Y Fro Gymraeg’: Ynys Môn, Gwynedd, Ceredigion and Carmarthenshire, particularly Gwynedd.

#### Class 4: English

This was the next largest group at 11% of the sample. Most identified as English and a few as British or Welsh. Very few reported speaking Welsh. This group was concentrated in the counties of Conwy, Denbighshire and Flintshire along the north coast.

#### Class 5: Ethnically Diverse

This was the smallest group, making up 4% of the sample. About a quarter identified as British, but few identified as Welsh or English. Very few reported speaking Welsh. Unlike the other four groups, which overwhelmingly reported ‘White Welsh/English/Scottish/Northern Irish/British’ ethnicity, this group reported a wide range of ethnicities. About 45% were White other. The next largest cluster (~ 24%) reported Asian ethnicities, with the remainder reporting Black, Arab or other ethnicities. They were highly concentrated in Cardiff, with smaller pockets in Swansea, Newport, Wrexham and Flintshire.

### Differences with Alternative Models

In the six- and seven-class models, a small ‘Cymrophone British’ class emerged, drawing from the Cymry Cymraeg and British groups. In the seven-class solution, the Ethnically Diverse group was divided into a group which was predominantly ‘White other’ in ethnicity and which did not identify as Welsh, British or English and a group which was largely people of colour, half of whom identified as British.

### Group Demographics

Table 1 also compares the groups on demographic, socio-economic and geographical factors. On age, two groups stood out: the Ethnically Diverse, who were younger than other groups, and the English, who were older. On education, the Ethnically Diverse had a strikingly high proportion of respondents with higher degrees and high rates of first degrees and foreign qualifications. The English and the Anglophone Welsh, in contrast, had lower rates of degree qualifications and higher rates of no qualifications. On income, the British and the Cymry Cymraeg were less likely to be in the lowest income bracket, while the British were more likely to be in the highest bracket, and the Ethnically Diverse group were over-represented at both extremes of the income scale. On material deprivation, the Cymry Cymraeg were less likely to be materially deprived while the Ethnically Diverse were more likely. There were no major differences in perceived financial pressure, except for a tendency for the Cymry Cymraeg to being more likely to report not having bills.

Geographically, the Ethnically Diverse and the Anglophone Welsh were over-represented in the most deprived quintile of LSOAs, while the Cymry Cymraeg were under-represented, and the British were over-represented in the least deprived quintile. The Cymry Cymraeg group was much more likely to live in the lowest density quintile of LSOAs, while the Ethnically Diverse group was less likely, with the reverse being the case for the highest density LSOAs.

### Analyses of Health

#### General Health

**Fig. 3:**
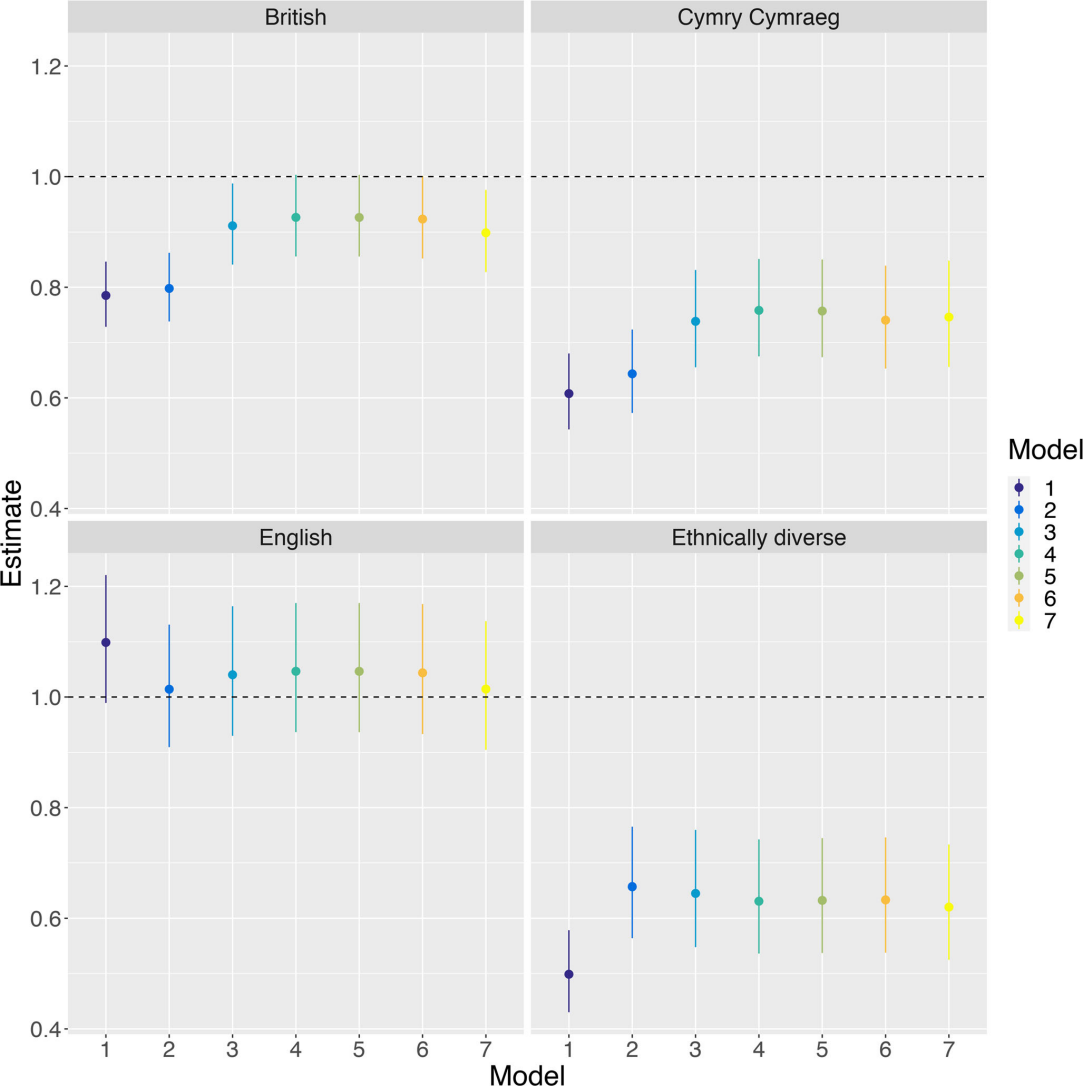
Odds ratios, with 95% confidence intervals, for reporting ‘not good’ general health for each group, relative to the Anglophone Welsh group, across all seven models

Table 2 and Fig. 3 display odds ratios (ORs) for reporting ‘not good’ health in the various models. Unadjusted, there are striking disparities in self-reported health: The Cymry Cymraeg, British and Ethnically Diverse groups had much lower rates of not good health than the Anglophone Welsh (reference) group, with the English looking similar to the Anglophone Welsh. These disparities were slightly attenuated in the adjusted models, with adjusting for age accounting for some of the reduced risk of the Ethnically Diverse group and the increased risk of the English, while accounting for education and income accounting for some of the reduced risk of the British and Cymry Cymraeg. Generally, however, risk remained lower for the British, Cymry Cymraeg and Ethnically Diverse groups, with the confidence intervals for the British group’s OR overlapping with 1 in some models, but the ORs for the Ethnically Diverse and Cymry Cymraeg groups remained comfortably below 1 across all models.

**Table 2:** Odds ratios and 95% confidence intervals for each term in each of the seven models for general health

Term	Model 1		Model 2		Model 3		Model 4		Model 5		Model 6		Model 7	
	OR	95% CI	OR	95% CI	OR	95% CI	OR	95% CI	OR	95% CI	OR	95% CI	OR	95% CI
British	0.79	0.73–0.85	0.80	0.74–0.86	0.91	0.84–0.99	0.93	0.86–1	0.93	0.86–1	0.92	0.85–1	0.9	0.83–0.98
Cymry Cymraeg	0.61	0.54–0.68	0.64	0.57–0.72	0.74	0.66–0.83	0.76	0.68–0.85	0.76	0.67–0.85	0.74	0.65–0.84	0.75	0.66–0.85
English	1.10	0.99–1.22	1.01	0.91–1.13	1.04	0.93–1.16	1.05	0.94–1.17	1.05	0.94–1.17	1.04	0.93–1.17	1.01	0.9–1.14
Ethnically Diverse	0.50	0.43–0.58	0.66	0.56–0.77	0.65	0.55–0.76	0.63	0.54–0.74	0.63	0.54–0.74	0.63	0.54–0.75	0.62	0.52–0.73
Age 25–34			1.35	1.18–1.54	1.67	1.45–1.92	1.62	1.41–1.86	1.62	1.41–1.86	1.62	1.41–1.86	1.41	1.22–1.62
Age 35–44			1.68	1.46–1.92	2.16	1.87–2.49	2.13	1.85–2.45	2.13	1.85–2.45	2.13	1.85–2.45	1.87	1.62–2.17
Age 45–54			2.10	1.85–2.38	2.55	2.23–2.92	2.55	2.23–2.92	2.55	2.23–2.92	2.55	2.23–2.92	2.4	2.09–2.75
Age 55–64			3.10	2.73–3.52	3.33	2.92–3.81	3.38	2.96–3.86	3.38	2.96–3.86	3.38	2.96–3.85	3.67	3.2–4.2
Age 65–74			3.77	3.32–4.28	3.65	3.19–4.19	3.77	3.29–4.32	3.77	3.29–4.32	3.76	3.28–4.31	4.79	4.16–5.5
Age 75+			6.14	5.37–7.02	5.20	4.5–6.01	5.46	4.72–6.31	5.45	4.72–6.3	5.45	4.71–6.3	7.25	6.25–8.41
Male			0.88	0.82–0.93	1.02	0.95–1.09	1.01	0.95–1.08	1.01	0.95–1.08	1.01	0.95–1.08	1.01	0.95–1.08
First degree					0.97	0.83–1.12	0.96	0.83–1.11	0.96	0.83–1.11	0.96	0.83–1.11	0.93	0.8–1.08
A/AS levels					1.15	0.97–1.36	1.13	0.95–1.33	1.13	0.95–1.33	1.13	0.95–1.33	1.09	0.92–1.29
Diplomas, etc.					1.56	1.34–1.81	1.50	1.29–1.75	1.50	1.29–1.75	1.50	1.29–1.75	1.4	1.2–1.63
O level/GCSE grades A–C, etc.					1.26	1.09–1.47	1.21	1.04–1.4	1.21	1.04–1.4	1.21	1.04–1.4	1.11	0.96–1.29
O level/GCSE grades D–G					1.84	1.5–2.27	1.71	1.39–2.1	1.71	1.39–2.1	1.70	1.38–2.1	1.5	1.21–1.86
Other qualifications					1.64	1.39–1.94	1.52	1.29–1.8	1.52	1.29–1.8	1.52	1.29–1.8	1.36	1.14–1.61
Trade apprenticeships					1.54	1.27–1.86	1.47	1.21–1.78	1.47	1.21–1.78	1.47	1.21–1.77	1.34	1.1–1.63
Foreign qualifications					1.67	1.05–2.66	1.50	0.95–2.39	1.50	0.95–2.39	1.50	0.94–2.38	1.55	0.97–2.48
No qualifications					2.29	1.97–2.67	2.07	1.78–2.42	2.07	1.78–2.42	2.07	1.78–2.41	1.77	1.52–2.07
£10,400 to £20,799/year					0.62	0.56–0.69	0.63	0.57–0.7	0.63	0.57–0.7	0.63	0.57–0.7	0.71	0.64–0.78
£20,800 to £31,099/year					0.41	0.35–0.48	0.42	0.36–0.49	0.42	0.36–0.49	0.42	0.36–0.49	0.52	0.45–0.61
£31,100 to £41,499/year					0.40	0.33–0.48	0.42	0.35–0.5	0.42	0.35–0.5	0.42	0.35–0.5	0.54	0.45–0.65
£41,500 or more/year					0.27	0.21–0.33	0.28	0.23–0.35	0.28	0.23–0.35	0.28	0.23–0.35	0.38	0.3–0.47
WIMD quintile 2							0.87	0.78–0.97	0.87	0.77–0.97	0.87	0.77–0.97	0.95	0.84–1.06
WIMD quintile 3							0.71	0.64–0.8	0.71	0.63–0.8	0.71	0.63–0.8	0.8	0.71–0.9
WIMD quintile 4							0.64	0.56–0.72	0.63	0.56–0.72	0.63	0.56–0.72	0.74	0.65–0.84
WIMD quintile 5							0.59	0.53–0.67	0.59	0.52–0.67	0.60	0.53–0.68	0.72	0.63–0.82
Density (z-scored)									0.99	0.95–1.03	1.00	0.96–1.04	1	0.96–1.04
Welsh Wales											1.06	0.94–1.18	1.07	0.96–1.2
Y Fro Gymraeg											1.12	0.96–1.3	1.07	0.92–1.25
Keeping up with all bills and commitments, but it is a struggle from time to time											2.29	2.03–2.58	1.41	1.3–1.54
Keeping up with all bills and commitments, but it is a constant struggle													1.93	1.67–2.24
Falling behind with some bills or credit commitments													2.31	1.79–2.98
Having real financial problems and have fallen behind with many bills or credit commitments													3.82	2.65–5.52
Have no bills													1.15	0.78–1.69
In material deprivation													2.23	2.01–2.47

#### Mental Health

**Fig. 4:**
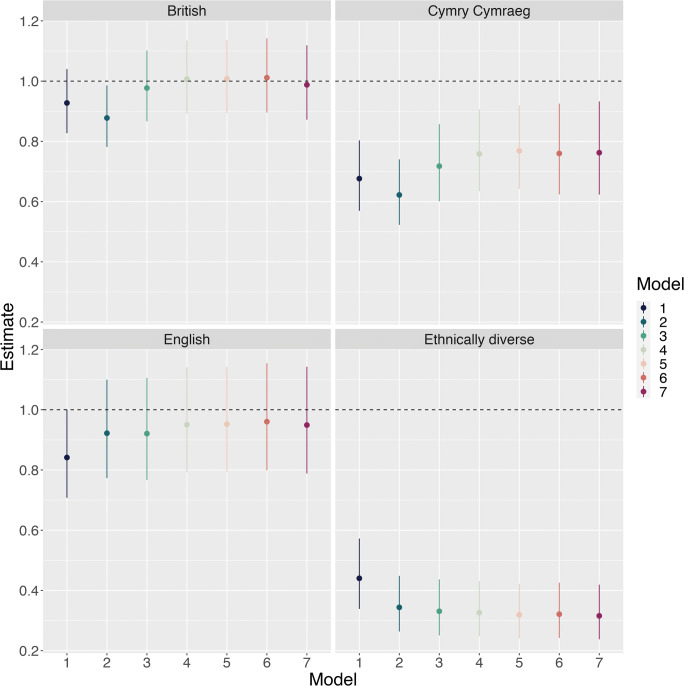
Odds ratios, with 95% confidence intervals, for reporting a mental health problem for each group, relative to the Anglophone Welsh group, across all seven models

Table 3 and Fig. 4 display ORs for reporting a mental health problem in the seven models. Again, the Cymry Cymraeg and, especially, the Ethnically Diverse groups had reduced risks in all models. The English had reduced risk in the unadjusted model, but adjusting for age gave them a similar risk to the Anglophone Welsh. The British had a decreased risk in model 2 only, suggesting their risk was low, considering their age and gender profile, but this was accounted for by the socio-economic variables.

**Table 3:** Odds ratios and 95% confidence intervals for each term in each of the seven models for mental health

Term	Model 1		Model 2		Model 3		Model 4		Model 5		Model 6		Model 7	
	OR	95% CI	OR	95% CI	OR	95% CI	OR	95% CI	OR	95% CI	OR	95% CI	OR	95% CI
British	0.93	0.83–1.04	0.88	0.78–0.99	0.98	0.87–1.1	1.01	0.89–1.14	1.01	0.89–1.14	1.01	0.9–1.14	0.99	0.87–1.12
Cymry Cymraeg	0.68	0.57–0.8	0.62	0.52–0.74	0.72	0.6–0.86	0.76	0.63–0.91	0.77	0.64–0.92	0.76	0.62–0.93	0.76	0.62–0.93
English	0.84	0.71–1	0.92	0.77–1.1	0.92	0.77–1.11	0.95	0.79–1.14	0.95	0.79–1.14	0.96	0.8–1.15	0.95	0.79–1.14
Ethnically Diverse	0.44	0.34–0.57	0.34	0.26–0.45	0.33	0.25–0.44	0.33	0.25–0.43	0.32	0.24–0.42	0.32	0.24–0.43	0.32	0.24–0.42
Age 25–34			1.11	0.95–1.31	1.42	1.2–1.68	1.36	1.15–1.62	1.37	1.16–1.62	1.37	1.16–1.62	1.15	0.97–1.37
Age 35–44			1.03	0.87–1.22	1.37	1.15–1.64	1.35	1.13–1.61	1.35	1.13–1.62	1.35	1.13–1.62	1.13	0.94–1.36
Age 45–54			0.90	0.76–1.06	1.12	0.94–1.33	1.12	0.94–1.33	1.13	0.95–1.34	1.13	0.95–1.34	0.98	0.82–1.18
Age 55–64			0.70	0.59–0.84	0.72	0.6–0.87	0.73	0.61–0.88	0.74	0.61–0.89	0.74	0.61–0.89	0.74	0.61–0.9
Age 65–74			0.29	0.24–0.36	0.26	0.21–0.33	0.27	0.22–0.35	0.28	0.22–0.35	0.28	0.22–0.35	0.36	0.28–0.45
Age 75+			0.21	0.16–0.27	0.16	0.12–0.21	0.17	0.13–0.23	0.17	0.13–0.23	0.17	0.13–0.23	0.24	0.18–0.32
Male			0.71	0.65–0.79	0.85	0.76–0.94	0.85	0.76–0.94	0.85	0.76–0.94	0.85	0.76–0.94	0.85	0.76–0.95
First degree					1.05	0.81–1.36	1.03	0.8–1.34	1.04	0.8–1.35	1.04	0.8–1.34	0.99	0.76–1.29
A/AS levels					1.14	0.87–1.51	1.11	0.84–1.45	1.10	0.84–1.45	1.10	0.84–1.45	1.04	0.79–1.37
Diplomas, etc.					1.34	1.03–1.75	1.28	0.98–1.66	1.28	0.99–1.67	1.28	0.98–1.67	1.14	0.87–1.49
O level/GCSE grades A–C, etc.					1.08	0.83–1.4	1.00	0.77–1.31	1.01	0.78–1.32	1.01	0.78–1.31	0.88	0.68–1.15
O level/GCSE grades D–G					1.59	1.15–2.18	1.43	1.04–1.96	1.44	1.05–1.98	1.45	1.05–1.99	1.19	0.86–1.65
Other qualifications					1.60	1.19–2.14	1.44	1.08–1.93	1.45	1.08–1.94	1.44	1.08–1.93	1.18	0.88–1.59
Trade apprenticeships					1.15	0.8–1.66	1.08	0.75–1.55	1.09	0.76–1.57	1.09	0.76–1.56	0.94	0.65–1.35
Foreign qualifications					1.10	0.43–2.81	0.97	0.38–2.5	0.98	0.38–2.53	0.98	0.38–2.53	0.98	0.37–2.59
No qualifications					2.14	1.65–2.79	1.88	1.44–2.44	1.89	1.45–2.46	1.89	1.45–2.45	1.42	1.08–1.86
£10,400 to £20,799/year					0.57	0.5–0.64	0.57	0.51–0.65	0.58	0.51–0.65	0.58	0.51–0.65	0.67	0.6–0.76
£20,800 to £31,099/year					0.30	0.25–0.37	0.31	0.26–0.38	0.32	0.26–0.38	0.32	0.26–0.38	0.42	0.35–0.51
£31,100 to £41,499/year					0.31	0.24–0.4	0.33	0.26–0.43	0.33	0.26–0.43	0.33	0.26–0.43	0.47	0.37–0.61
£41,500 or more/year					0.17	0.12–0.24	0.18	0.12–0.26	0.18	0.13–0.26	0.18	0.13–0.26	0.27	0.18–0.39
WIMD quintile 2							0.86	0.72–1.03	0.87	0.73–1.04	0.88	0.73–1.05	0.97	0.81–1.17
WIMD quintile 3							0.70	0.58–0.84	0.71	0.59–0.86	0.72	0.59–0.87	0.82	0.68–0.99
WIMD quintile 4							0.56	0.46–0.68	0.57	0.47–0.7	0.58	0.48–0.71	0.71	0.58–0.87
WIMD quintile 5							0.53	0.44–0.65	0.55	0.45–0.67	0.56	0.45–0.69	0.7	0.57–0.87
Density (z-scored)									1.04	0.98–1.1	1.05	0.99–1.11	1.04	0.99–1.11
Welsh Wales											1.08	0.94–1.24	1.1	0.96–1.27
Y Fro Gymraeg											1.09	0.88–1.33	1.03	0.84–1.27
Keeping up with all bills and commitments, but it is a struggle from time to time											2.29	2.03–2.58	1.37	1.2–1.56
Keeping up with all bills and commitments, but it is a constant struggle													1.67	1.37–2.05
Falling behind with some bills or credit commitments													2.15	1.6–2.89
Having real financial problems and have fallen behind with many bills or credit commitments													4.61	3.21–6.61
Have no bills													1.5	0.82–2.75
In material deprivation													2.4	2.09–2.76

### Post hoc Analyses

Given that the Ethnically Diverse group might hide heterogeneity between different ethnic groups, modified versions of model 1 for general and mental health were fitted to just the members of this group, with fixed effects of self-reported ethnicity replacing those of group. As the largest group, White other was used as the reference category.

#### General Health

With the exception of the Mixed group, all OR confidence intervals overlapped with 1.00 ORs for the Asian (1.24, 0.78–1.97), Black (OR = 1.05, 1.51–2.15), White Welsh/English/Scottish/Northern Irish/British (OR = 1.54, 0.64–3.71) and Other (OR = 1.31, 0.73–2.34) respondents in the Ethnically Diverse group trended towards poorer health, while the Mixed ethnicity respondents reported strikingly poorer health (OR = 4.60, 1.08–19.56).

#### Mental Health

Asian members of the Ethnically Diverse group reported better mental health than the reference group (OR = 0.25, 0.07–0.90). Point estimates of risk were lower for Black respondents, but CIs overlapped substantially with 1 (OR = 0.35, 0.04–2.90). Mixed (OR = 2.42, 0.04–126.45), White Welsh/English/Scottish/Northern Irish/British (OR = 2.98, 0.50–17.78) and Other (OR = 1.64, 0.49–5.47) respondents had lower point estimates for mental health risk, but again, all CIs overlapped substantially with 1.

It should be noted that sample sizes within this analysis were very low, making it very plausible that some of these non-significant associations are false negatives. It should also be noted that the majority of Mixed ethnicity respondents were assigned to groups other than the Ethnically Diverse group by the latent class analysis, and those respondents in this group should not be assumed to be representative of Mixed ethnicity people in Wales.

## Discussion

The present paper demonstrates striking health disparities between the various national identity groups of Wales, which are not explained by obvious socio-demographic or geographic differences between the groups.

Latent class analysis identified five identity groups in Wales. Three map approximately, with caveats, onto Balsom’s Three-Wales Model, while two are novel. The Anglophone Welsh are a much broader group than Balsom’s Welsh Wales. They comprise a plurality across most of the country, including in counties that Balsom identified as British Wales, such as Wrexham and Pembrokeshire. Despite this broader conception, they remained educationally disadvantaged compared to the Cymry Cymraeg, Ethnically Diverse and British, although not the English, and more likely to be materially deprived than the Cymry Cymraeg and British, although at lower risk than the Ethnically Diverse.

The British were distributed quite differently to Balsom’s model, with the distribution looking strikingly similar to that of the English, but more in rural and less deprived areas. Although they were geographically co-located with the English, they tended to have higher levels of education and were more likely to have higher levels of incomes. It is tempting to speculate that both groups represent English-born residents of Wales, with the distinction between them being largely one of socio-economic classes, echoing previous work showing a class dimension to English/British identification [40]. However, given that these two groups represented nearly 40% of the sample and the UK Census suggested that only 20.8% of the Welsh population was born in England (and many of them identify as Welsh), this does not seem likely. Although English-born residents were more likely to identify in the census as British (including combinations like Welsh and British) than Welsh-born residents (41% compared to 21%), the majority of people identifying as British are Welsh-born.

The Cymry Cymraeg closely resembled Y Fro Gymraeg from the Three-Wales Model, characterised by their use of the Welsh language, tendency to live in the Y Fro Gymraeg counties (although not exclusively) and identification as Welsh. This group had greater rates of higher pay and advanced qualifications than the Anglophone Welsh. They also tended not to live in deprived areas. It should be noted that the Welsh language variable in the latent class analysis measured *use* of Welsh, rather than just the ability to speak it. This likely led to a smaller and more specific Cymry Cymraeg group than a more inclusive definition based on ability, with substantial minorities of those able to speak Welsh falling into the Anglophone Welsh and British groups. This paper, thus, perhaps takes the implicit stance that a language’s importance to identity comes from its practice, rather than simply ability.

The English were not featured in the Three-Wales Model, but, as mentioned above, they are clearly distinct from the British in terms of national identity and socio-economic status. As mentioned above, they looked somewhat like the British in terms of geographical distribution, albeit in more deprived LSOAs and more focused on the north coast than the British. They were the oldest group and were relatively financially disadvantaged. Data were not available on country of birth, but it is likely that most of this group was born in England and migrated to Wales.

Finally, the Ethnically Diverse group was the second addition. As well as the obvious heterogeneity in ethnicity, they also, strikingly, had the highest proportion of any group in both the highest and lowest income bands. There were, however, commonalities. They were by some distance the most highly educated, youngest and most urban group. While the English would likely been part of British Wales in the Three-Wales Model, the Ethnically Diverse group feel like an entirely novel addition. It is worth reiterating that this group should not be read as a ‘people of colour group’: The plurality of the group fall under the ‘White Other’ group in the classification system used in UK official data.

The ‘Five-Wales model’ used here and Balsom’s Three-Wales Model have clear similarities, but also important differences. Firstly, although the present model incorporates geographical information, it is people, rather than areas, that are classified. The Three-Wales Model conversely classifies regions, treating their residents as monolithic groups. Thus, not only is the present model is thus able to identify less populous groups, but it is also less vulnerable to the ecological fallacy when used in practice. Secondly, the present approach employs a data-driven approach to classification, while the Three-Wales Model draws on an attempt to synthesise what Raymond Williams called Wales’ ‘two truths’ [12]: the industrial labourist tradition of South Wales and the Welsh-speaking culture of Y Fro Gymraeg. While the latter certainly captures something of the ways that Wales has been depicted culturally and artistically, the present approach is perhaps more suitable for empirical research.

In terms of general health, the Anglophone Welsh and English had the worst outcomes, with the British trending towards slightly better health, depending on the model in question, and the Cymry Cymraeg and Ethnically Diverse groups reporting much better outcomes. For mental health, similar results were found, except that the British looked more similar to the Anglophone Welsh, and the reduced risk in the Ethnically Diverse group was even more pronounced.

### Possible Mechanisms

As outlined in the “Introduction”, there were clear reasons to expect that the Anglophone Welsh group would have poorer health than the other groups. That being said, as this group was far broader than its more valleys-focused counterpart in Balsom’s Three-Wales Model, explanations based solely on post-industrial health risks are likely insufficient. Models 3–7 accounted for various components of socio-economic status and large health disparities largely endured. Although it is likely that some of the remaining disparities represented residual confounding, the remaining disparities are large enough that other factors are likely also responsible. Indeed, the associations between the identity groups and health remain reasonably stable after model 3, when education and income are included, even when other measures of poverty are added, which seems inconsistent with an explanation solely based on residual confounding by socio-economic status.

The Cymry Cymraeg group had, as expected, lower rates of health and mental health problems than the Anglophone Welsh group, results that are only partly explained by differences in socio-economic status. The results are reminiscent of work comparing the Finnish-speaking majority and Swedish-speaking minority in Finland [41], where the better health of Swedish minority is partly attributable to greater social capital. This is also a possibility in Wales, where geographical variability in social capital favours some of the rural areas where Welsh speakers predominantly live, and is particularly low in the South Wales Valleys [20, 21]. More broadly, degree of cultural assimilation or cultural distinctiveness has been shown to be related to health in Japanese-Americans [23] and, potentially, these differences represent something similar.

The group with the lowest levels of poor health was the Ethnically Diverse. This is perhaps a surprising finding, given that in the broad literature on ethnic health disparities, it is more common for such disparities to favour ethnic majorities (e.g. [42]). It is also important to highlight that other work in Wales has found health disparities to the detriment of people of colour, such as during the COVID-19 pandemic [43]. However, it is again important to remember that the plurality of this group fell into the ‘White other’ category. Post hoc analyses found some evidence for heterogeneity of outcomes by ethnicity within the Ethnically Diverse group, but given the small size of the subgroups in question, further work is needed on this topic.

Although data on country of birth were not available, it may be that a healthy migrant effect [44] accounts for some of the Ethnically Diverse group’s health advantage. This contrasts with the English, the other group where one might speculate that a substantial proportion is migrants to Wales, who had much poorer health. Health selection effects by migration, previously identified elsewhere in Wales [45], likely depend on the reasons for migration, which, in turn, likely vary geographically. The Ethnically Diverse group is concentrated in Cardiff, a major city, while the English are concentrated along the north coast, a popular retirement location. Further work is needed to characterise this apparent geographic heterogeneity in health selection effects.

### Limitations

The analyses have a number of limitations and caveats that are worth highlighting. Firstly, the use of latent class analysis could be questioned—Why not simply model the health outcomes of those identifying with different nationalities? Such an approach would have had important limitations. Firstly, it would have conflated the Anglophone Welsh and Cymry Cymraeg groups, both ignoring the previous work showing these groups to be meaningfully distinct [9, 10] and missing the health inequalities between these groups. Secondly, this would not have integrated the information on geography into the model, which previous work has shown is important for Welsh identity. The use of latent class analysis, conversely, allowed the model to be informed by previous work, while still allowing it to deviate where this was a poor fit for the data—for example the broader conception of the Anglophone Welsh group than Balsom’s Welsh Wales construct. That being said, it is important to reiterate that the five-class model is used as a useful heuristic, rather than being proposed as the definitive model of Welsh identity. As a case in point, the six- and seven-class models included further plausible classes—the Welsh-speaking British and a division of the Ethnically Diverse group into a substantially British-identifying group of people of colour and a group who did not identify with any of the provided national identities, with predominantly ‘White other’ ethnicities.

One could also question the choice of variables that were used in the latent class analysis. A notable exception was class. Here, the decision was made to use proxies for class in the second stage of analysis to assess the extent to which group differences could be explained by class, and for it to feature at both stages of analysis, it seemed to have the potential for circularity—defining groups (partly) by class and then seeing whether the differences between them could be explained by class. Another potentially contentious decision was using a variable measuring respondents’ use of the Welsh language, rather than just the ability to speak it, as mentioned above.

Another possible criticism is that the Ethnically Diverse group represented a ‘none of the above’ category, rather than a coherent group. It is certainly clear that this group does not represent a single national identity. That being said, this approach can be justified on a number of grounds. Firstly, there were no good alternatives. Excluding this group or attempting to merge it into one of the other groups would have been both clearly unsatisfactory and ad hoc. Furthermore, a limited number of response options were available for the questions concerning national identity, so it would not have been possible to make some of these distinctions using the available data anyway. Some exploratory post hoc analyses were run within the group between different ethnic identities, but these were underpowered due to the limited sample size. More positively, the comparisons between the Ethnically Diverse group and the others reveal some striking and interesting health disparities. Furthermore, although this group was clearly heterogenous in terms of ethnicity, and probably in terms of national identity, there were also commonalities, namely youth, high education and urbanicity.

Finally, it should be emphasised that this is a piece of descriptive epidemiology, and any speculation about the causal mechanisms that underlie these health disparities is speculative. Identifying causal effects in a context like this is challenging, but possible [25, 46], and will hopefully be the subject of future work.

## Conclusion

National identity has been a surprising absentee from social epidemiology research—Identities, ethnic and civic, have clear relationships to health. This work confirms this empirically, finding wide disparities among five national identity groups in Wales. Wales is an ideal venue for this research, as part of a multi-national state where national identity is ambiguous and negotiated, but is likely representative of many other nations in this regard. National identity remains a powerful social force in the twenty-first century, and health is part of that story.

## Supplementary Information

ESM 1 (DOCX 24 kb)

## Data Availability

Data are property of the Welsh Government. Non-identifying data are available through the UK Data Service (https://www.ukdataservice.ac.uk/). More detailed data on respondents’ areas of residence and ethnicities were provided by the Welsh Government’s survey team (Surveys@gov.wales) under a data access agreement.
