# Supplementary material for: Health and Mental Health Disparities Between National Identity Groups in Wales
Source: J Racial Ethn Health Disparities. 2021 Jan 19;9(1):270–87. doi: 10.1007/s40615-020-00951-z (PMC7815193; doi:10.1007/s40615-020-00951-z)
Supplement: Supplementary file 1 — (DOCX 24 kb) [file 40615_2020_951_MOESM1_ESM.docx]

# Here is the code for the attached manuscript.

# The data are not mine to share and you will need a data access agreement

# with the Welsh Government to access some of the data. The relevant people

#can be contacted on Surveys@gov.wales and I'm happy to share a copy of the

#data access if that would be useful. Apologogies for my clunky code!

set.seed(123)

library(dplyr)

library(glmmTMB)

library(performance)

library(ggplot2)

library(sf)

library(pals)

library(foreign)

library(patchwork)

library(dvmisc)

library(poLCA)

library(Amelia)

library(broom.mixed)

library(tableone)

sessionInfo()

# R version 4.0.2 (2020-06-22)

# Platform: x86_64-apple-darwin17.0 (64-bit)

# Running under: macOS Mojave 10.14.6

#

# Matrix products: default

# BLAS: /System/Library/Frameworks/Accelerate.framework/Versions/A/Frameworks/vecLib.framework/Versions/A/libBLAS.dylib

# LAPACK: /Library/Frameworks/R.framework/Versions/4.0/Resources/lib/libRlapack.dylib

#

# Random number generation:

# RNG: Mersenne-Twister

# Normal: Inversion

# Sample: Rounding

#

# locale:

# [1] en_GB.UTF-8/en_GB.UTF-8/en_GB.UTF-8/C/en_GB.UTF-8/en_GB.UTF-8

#

# attached base packages:

# [1] stats graphics grDevices utils datasets methods base

#

# other attached packages:

# [1] tableone_0.12.0 merTools_0.5.2 arm_1.11-2 lme4_1.1-23 Matrix_1.2-18 broom.mixed_0.2.6

# [7] Amelia_1.7.6 Rcpp_1.0.5 poLCA_1.4.1 MASS_7.3-51.6 scatterplot3d_0.3-41 dvmisc_1.1.4

# [13] rbenchmark_1.0.0 patchwork_1.0.1 foreign_0.8-80 pals_1.6 sf_0.9-6 ggplot2_3.3.2

# [19] performance_0.5.0 glmmTMB_1.0.2.1 dplyr_1.0.2

#

# loaded via a namespace (and not attached):

# [1] minqa_1.2.4 colorspace_1.4-1 ellipsis_0.3.1 class_7.3-17 estimability_1.3 htmlTable_2.1.0

# [7] base64enc_0.1-3 dichromat_2.0-0 rstudioapi_0.11 farver_2.0.3 fansi_0.4.1 mvtnorm_1.1-1

# [13] codetools_0.2-16 splines_4.0.2 knitr_1.30 Formula_1.2-4 nloptr_1.2.2.2 broom_0.7.0

# [19] cluster_2.1.0 tab_4.1.1 png_0.1-7 shiny_1.5.0 mapproj_1.2.7 compiler_4.0.2

# [25] emmeans_1.5.1 backports_1.1.10 assertthat_0.2.1 fastmap_1.0.1 survey_4.0 cli_2.0.2

# [31] later_1.1.0.1 htmltools_0.5.0 tools_4.0.2 coda_0.19-4 gtable_0.3.0 glue_1.4.2

# [37] reshape2_1.4.4 maps_3.3.0 vctrs_0.3.4 nlme_3.1-148 iterators_1.0.12 insight_0.9.6

# [43] xfun_0.17 stringr_1.4.0 mime_0.9 lifecycle_0.2.0 statmod_1.4.34 zoo_1.8-8

# [49] scales_1.1.1 hms_0.5.3 promises_1.1.1 TMB_1.7.18 RColorBrewer_1.1-2 gridExtra_2.3

# [55] labelled_2.7.0 rpart_4.1-15 latticeExtra_0.6-29 stringi_1.5.3 bayestestR_0.7.2 foreach_1.5.0

# [61] e1071_1.7-3 blme_1.0-4 checkmate_2.0.0 boot_1.3-25 rlang_0.4.7 pkgconfig_2.0.3

# [67] lattice_0.20-41 purrr_0.3.4 htmlwidgets_1.5.1 labeling_0.3 tidyselect_1.1.0 plyr_1.8.6

# [73] magrittr_1.5 R6_2.4.1 generics_0.0.2 Hmisc_4.4-1 DBI_1.1.0 pillar_1.4.6

# [79] haven_2.3.1 withr_2.3.0 units_0.6-7 survival_3.1-12 abind_1.4-5 nnet_7.3-14

# [85] tibble_3.0.3 crayon_1.3.4 KernSmooth_2.23-17 jpeg_0.1-8.1 grid_4.0.2 data.table_1.13.0

# [91] forcats_0.5.0 digest_0.6.25 classInt_0.4-3 xtable_1.8-4 tidyr_1.1.2 httpuv_1.5.4

# [97] munsell_0.5.0 mitools_2.4

#DataFolder is an encrypted local drive I keep potentially sensitive data on

#thisFolder is a Dropbox folder with scripts and other less sensitive stuff

setwd(DataFolder)

# Read in publically accessable data

read.spss('nsw_2017-18_respondent_file_final_ukds.sav', to.data.frame = T, stringsAsFactors = F) -> NSfW1718

read.spss('national_survey_for_wales_2018-19_respondent_file_ukds.sav', to.data.frame = T, stringsAsFactors = F) -> NSfW1819

# Read in location and ethnicity data

read.spss('2017-18.sav', to.data.frame = T, stringsAsFactors = F) -> NSfW1718_Locs

read.spss('2018-19.sav', to.data.frame = T, stringsAsFactors = F) -> NSfW1819_Locs

setwd(thisFolder)

# Tidy up and merge data

trimws(NSfW1718_Locs $DvLSOA2011) -> NSfW1718_Locs $DvLSOA2011

trimws(NSfW1819_Locs $DvLSOA2011) -> NSfW1819_Locs $DvLSOA2011

merge(NSfW1718, NSfW1718_Locs, by = 'CaseNo', all.x = T) -> NSfW1718

merge(NSfW1819, NSfW1819_Locs, by = 'CaseNo', all.x = T) -> NSfW1819

#Gather relevant data from both years and bind

NSfW1718 $Ethnicity <- NSfW1718 $Ethnicity.y

dplyr::select(NSfW1718, CaseNo, NatIdWel, NatIdWel, NatIdEng, NatIdBrit, Gender, WelSpk, GenHealth, Dvillchap3, SampleAdultWeight.x, DvLSOA2011, DvLA, Ethnicity, Educat, DvMatDep, DvFinBilCred, IncResp, WelFrqSpk, DvAgeGrp7, DvWIMDOvr5) -> NSfW1718x

NSfW1819 $Ethnicity <- NSfW1819 $Ethnicity.y

dplyr::select(NSfW1819, CaseNo, NatIdWel, NatIdWel, NatIdEng, NatIdBrit, Gender, WelSpk, GenHealth, Dvillchap3, SampleAdultWeight.x, DvLSOA2011, DvLA, Ethnicity, Educat, DvMatDep, DvFinBilCred, IncResp, WelFrqSpk, DvAgeGrp7, DvWIMDOvr5) -> NSfW1819x

rbind(NSfW1718x, NSfW1819x) -> NSfW1719

#Add 'non-speaker' level to frequency of spoken Welsh variable

as.character(NSfW1719 $WelFrqSpk) -> NSfW1719 $WelshFreq

NSfW1719 $WelshFreq[is.na(NSfW1719 $WelshFreq)] <- 'Non-speaker'

factor(NSfW1719 $WelshFreq) -> NSfW1719 $WelshFreq

setwd(DataFolder)

#Read in some data on LSOA level: area density (according to census)

read.csv('LSOA_AreaData.csv', head=T) -> LSOA_AreaData

dplyr::select(LSOA_AreaData, LSOAName, LSOACode, Density) -> LSOA_AreaData

merge(NSfW1719, LSOA_AreaData, by.x = 'DvLSOA2011', by.y = 'LSOACode', all.x = T) -> NSfW1719x

setwd(thisFolder)

# Dichotomise general health

NSfW1719x $GenHealthD [NSfW1719x $GenHealth%in% c('Fair', 'Bad', 'Very bad')] <- 'Yes'

NSfW1719x $GenHealthD [NSfW1719x $GenHealth%in% c('Very good', 'Good')] <- 'No'

factor(NSfW1719x$GenHealthD) -> NSfW1719x$GenHealthD

#Tidy up education variable

NSfW1719x $Educat[NSfW1719x $Educat=="None of these / No qualifications"] <- "No qualifications"

NSfW1719x$LSOAName <- factor(NSfW1719x$LSOAName)

NSfW1719x$DvLSOA2011 <- factor(NSfW1719x$DvLSOA2011)

######

#Aggregate ethnic groups into six category system (Amelia does not accept nominal variables with >10 levels)

NSfW1719x$EthnicityGrouped[NSfW1719x$Ethnicity %in% c('White - Welsh/English/Scottish/Northern Irish/British' )] <- 'WhiteWelshBritishEnglish'

NSfW1719x$EthnicityGrouped[NSfW1719x$Ethnicity %in% c('White - Other', 'White - Polish (SPONTANEOUS ONLY)', 'White - Irish', 'White - Gypsy or Irish Traveller')] <- 'WhiteOther'

NSfW1719x$EthnicityGrouped[NSfW1719x$Ethnicity %in% c('Black - African', 'Black - Caribbean', 'Black - Other')] <- 'Black'

NSfW1719x$EthnicityGrouped[NSfW1719x$Ethnicity %in% c('Asian - Indian', 'Asian - Pakistani', 'Asian - Bangladeshi', 'Asian - Chinese', 'Asian - Other')] <- 'Asian'

NSfW1719x$EthnicityGrouped[NSfW1719x$Ethnicity %in% c('Mixed - White and Black Caribbean', 'Mixed - White and Black African', 'Mixed - White and Asian', 'Mixed - Other')] <- 'Mixed'

NSfW1719x$EthnicityGrouped[NSfW1719x$Ethnicity %in% c('Arab', 'Other', 'Other - Any other ethnic group', 'Other - Arab')] <- 'Other'

factor(NSfW1719x$EthnicityGrouped) -> NSfW1719x$EthnicityGrouped

#Create Three-Wales Model regions variable

NSfW1719x$ThreeWalesRegion[NSfW1719x$DvLA %in% c("Isle of Anglesey", "Gwynedd", "Ceredigion", "Carmarthenshire")] <- 'Y Fro Gymraeg'

NSfW1719x$ThreeWalesRegion[NSfW1719x$DvLA %in% c("Conwy", "Denbighshire", "Flintshire","Pembrokeshire", "Wrexham", "Powys", "Monmouthshire", "Newport", "Vale of Glamorgan", "Bridgend", "Cardiff" )] <- 'British Wales'

NSfW1719x$ThreeWalesRegion[NSfW1719x$DvLA %in% c("Swansea","Neath Port Talbot","Rhondda Cynon Taf", "Merthyr Tydfil","Caerphilly","Blaenau Gwent","Torfaen")] <- 'Welsh Wales'

NSfW1719x$ThreeWalesRegion <- factor(NSfW1719x$ThreeWalesRegion)

## First multiple imputation (for LCA)

##########

# Declare variables to include in imputation

Nominals <- c('Gender', 'Educat', 'DvMatDep', 'DvFinBilCred', 'NatIdWel', 'NatIdEng', 'NatIdBrit', 'GenHealthD', 'Dvillchap3', 'EthnicityGrouped', 'ThreeWalesRegion')

Ordinals <- c( 'IncResp', 'DvWIMDOvr5', 'WelshFreq')

IDS <- c('CaseNo', 'DvLA', 'LSOAName', 'DvLSOA2011')

Variables <- c(Nominals, Ordinals, IDS)

# Impute (one imputation)

impute.out <- amelia(NSfW1719x[,Variables], idvars = IDS,noms =Nominals , ords = Ordinals, m = 1)

impute.out$imputations[[1]] -> ImputedForLCA

#########

#Run LCAs.

set.seed(123)

poLCA(cbind(WelshFreq, DvLA, NatIdWel, NatIdBrit, NatIdEng, EthnicityGrouped)~1, nclas = 1, maxiter = 100000, graphs=T, nrep = 20, data= ImputedForLCA) -> LCA1

poLCA(cbind(WelshFreq, DvLA, NatIdWel, NatIdBrit, NatIdEng, EthnicityGrouped)~1, nclas = 2, maxiter = 100000, graphs=T, nrep = 20, data= ImputedForLCA) -> LCA2

poLCA(cbind(WelshFreq, DvLA, NatIdWel, NatIdBrit, NatIdEng, EthnicityGrouped)~1, nclas = 3, maxiter = 100000, graphs=T, nrep = 20, data= ImputedForLCA) -> LCA3

poLCA(cbind(WelshFreq, DvLA, NatIdWel, NatIdBrit, NatIdEng, EthnicityGrouped)~1, nclas = 4, maxiter = 100000, graphs=F, nrep = 20, data= ImputedForLCA) -> LCA4

poLCA(cbind(WelshFreq, DvLA, NatIdWel, NatIdBrit, NatIdEng, EthnicityGrouped)~1, nclas = 5, maxiter = 100000, graphs=F, nrep = 20, data= ImputedForLCA) -> LCA5

poLCA(cbind(WelshFreq, DvLA, NatIdWel, NatIdBrit, NatIdEng, EthnicityGrouped)~1, nclas = 6, maxiter = 100000, graphs=F, nrep = 20, data= ImputedForLCA) -> LCA6

poLCA(cbind(WelshFreq, DvLA, NatIdWel, NatIdBrit, NatIdEng, EthnicityGrouped)~1, nclas = 7, maxiter = 100000, graphs=F, nrep = 20, data= ImputedForLCA) -> LCA7

poLCA(cbind(WelshFreq, DvLA, NatIdWel, NatIdBrit, NatIdEng, EthnicityGrouped)~1, nclas = 8, maxiter = 100000, graphs=F, nrep = 20, data= ImputedForLCA) -> LCA8

poLCA(cbind(WelshFreq, DvLA, NatIdWel, NatIdBrit, NatIdEng, EthnicityGrouped)~1, nclas = 9, maxiter = 100000, graphs=F, nrep = 20, data= ImputedForLCA) -> LCA9

#Make Figure 1 (Scree plot of BICs)

ModelBICs <- data.frame(Model = factor(paste("Model", 1:9)), BIC=c(LCA1$bic, LCA2$bic, LCA3$bic, LCA4$bic, LCA5$bic, LCA6$bic, LCA7$bic, LCA8$bic, LCA9$bic))

tiff("BIC_Scree.tiff", width = 6, height = 6, units = 'in', res = 300, compression = 'lzw', type='cairo')

ggplot(aes(x = Model, y = BIC), data = ModelBICs) + geom_point() + theme(axis.text.x = element_text(angle = 270, vjust = 0.5, hjust=1)) + labs(x = '')

dev.off()

# Attach classes to unimputed data

LCA5 $predclass -> NSfW1719x $LCA5

NSfW1719x $LCA5 <- factor(NSfW1719x $LCA5, labels = c('English', 'British','Ethnically diverse', 'Cymry Cymraeg', 'Anglophone Welsh'))

LCA7 $predclass -> NSfW1719x $LCA7

NSfW1719x $LCA7 <- factor(NSfW1719x $LCA7, labels = c('English', 'Anglophone Welsh','People of colour', 'Cymrophone Welsh', 'Anglophone British', 'Non-UK', 'Cymrophone British'))

#Write out

setwd(DataFolder)

write.table(NSfW1719x, 'WalesNationalIdentity.csv', sep = ',', row.names = F)

setwd(thisFolder)

### Once the bit above has been run one, script can begin here in future

setwd(DataFolder)

read.csv('WalesNationalIdentity.csv') -> NSfW1719x

read.csv('LSOA_AreaData.csv', head=T) -> LSOA_AreaData

setwd(thisFolder)

#Make sure reference categories correct for all variables

factor(NSfW1719x$LCA5, levels = c( "Anglophone Welsh","British", "Cymry Cymraeg", "English", "Ethnically diverse")) -> NSfW1719x$LCA5

factor(NSfW1719x$LCA7, levels = c("Anglophone Welsh", "Anglophone British", "Cymrophone Welsh", "English", "Cymrophone British", "People of colour", "Non-UK")) -> NSfW1719x$LCA7

factor(NSfW1719x$Educat, levels = c( "Higher degree / postgraduate qualifications", "First degree", "A/AS levels","Diplomas, etc.", "O Level / GCSE grades A-C, etc.","O Level / GCSE grades D-G", "Other qualifications", "Trade apprenticeships", "Foreign qualifications", "No qualifications", "None of these / No qualifications")) -> NSfW1719x$Educat

factor(NSfW1719x$IncResp, levels= c( "Less than £10,400 a year","£10,400 to £20,799 a year", "£20,800 to £31,099 a year", "£31,100 to £41,499 a year", "£41,500 or more a year")) -> NSfW1719x$IncResp

factor(NSfW1719x$DvMatDep, levels = c('Not in material deprivation', 'In material deprivation')) -> NSfW1719x$DvMatDep

factor(NSfW1719x$DvFinBilCred, levels = c("Keeping up with all bills and commitments without any difficulties" , "Keeping up with all bills and commitments but it is a struggle from time to time","Keeping up with all bills and commitments but it is a constant struggle", "Falling behind with some bills or credit commitments", "Having real financial problems and have fallen behind with many bills or credit commitments", "Have no bills")) -> NSfW1719x$DvFinBilCred

factor(NSfW1719x$DvAgeGrp7) -> NSfW1719x$DvAgeGrp7

factor(NSfW1719x$Gender) -> NSfW1719x$Gender

factor(NSfW1719x$Educat) -> NSfW1719x$Educat

factor(NSfW1719x$NatIdWel, levels = c('Not selected', 'Welsh')) -> NSfW1719x$NatIdWel

factor(NSfW1719x$NatIdBrit, levels = c('Not selected', 'British')) -> NSfW1719x$NatIdBrit

factor(NSfW1719x$NatIdEng, levels = c('Not selected', 'English')) -> NSfW1719x$NatIdEng

factor(NSfW1719x$ThreeWalesRegion) -> NSfW1719x$ThreeWalesRegion

factor(NSfW1719x$DvWIMDOvr5, levels = c( 'Most deprived 20%', 'Q2', 'Q3', 'Q4', 'Least deprived 20%')) -> NSfW1719x$DvWIMDOvr5

factor(NSfW1719x$WelshFreq) -> NSfW1719x$WelshFreq

factor(NSfW1719x$EthnicityGrouped, levels = c('WhiteOther', 'Asian', 'Black', 'Mixed', 'Other', 'WhiteWelshBritishEnglish')) -> NSfW1719x$EthnicityGrouped

factor(NSfW1719x$Dvillchap3) -> NSfW1719x$Dvillchap3

factor(2-as.numeric(NSfW1719x$Dvillchap3), labels = c('No','Yes')) -> NSfW1719x$MH

factor(NSfW1719x$GenHealthD, labels = c('Good', 'Not good') ) -> NSfW1719x$GenHealthD

quant_groups(NSfW1719x$Density, 5) -> NSfW1719x$DensityQuant

#Make Table 1

write.csv(print(CreateTableOne(data=dplyr::select(NSfW1719x, GenHealthD, MH, NatIdWel, NatIdEng, NatIdBrit, EthnicityGrouped, WelshFreq, Gender, DvAgeGrp7, Educat, IncResp, DvWIMDOvr5, DensityQuant, DvMatDep, DvFinBilCred, ThreeWalesRegion, DvLA, LCA5), strata = 'LCA5')), 'WelshIdentityTable1.csv')

#Make the maps in figure 2

data.frame(matrix(table(NSfW1719x$DvLA, NSfW1719x$LCA5), ncol = 5)) -> GroupByLA

NSfW1719x$DvLA <- factor(NSfW1719x$DvLA)

GroupByLA $LA <- levels(NSfW1719x$DvLA)

colnames(GroupByLA) <- c( 'AnglophoneWelsh', 'British', 'CymryCymraeg', 'English', 'EthnicallyDiverse', 'LAName')

rowSums(GroupByLA[1:5]) -> GroupByLA$N

merge(LSOA_AreaData, GroupByLA, by = 'LAName', all.x = T) -> LSOA_AreaData1

setwd(DataFolder)

#Shapefile for local authorities

st_read("wales_lad_2011.shp") -> ShapesW

setwd(thisFolder)

fortify(ShapesW, region= 'code') -> ShapesWsf

GroupByLA$LA[GroupByLA $LA=='Vale of Glamorgan'] <- 'The Vale of Glamorgan'

mergedWales <-merge(ShapesWsf, GroupByLA, by.x="name", by.y="LA")

ggplot() + geom_sf(data = mergedWales, aes(fill = AnglophoneWelsh/N), lwd = 0) + coord_sf(crs = st_crs(mergedWales), datum = NA)+ scale_fill_gradientn(colours =(brewer.reds(20)), name = 'Anglophone Welsh ', breaks = c(.05,.25,.45,.65)) + theme( text = element_text(size = 20, family = 'sans', colour = 'black'), panel.grid = element_blank(), rect = element_blank(), plot.background = element_rect(fill = "white")) -> MapAnglophoneWelsh

ggplot() + geom_sf(data = mergedWales, aes(fill = British/N), lwd = 0) + coord_sf(crs = st_crs(mergedWales), datum = NA)+ scale_fill_gradientn(colours = rev(ocean.ice(20)), name = 'British ', breaks = c(.20,.25,.30, .35)) + theme( text = element_text(size = 20, family = 'sans', colour = 'black'), panel.grid = element_blank(), rect = element_blank(), plot.background = element_rect(fill = "white")) -> MapBritish

ggplot() + geom_sf(data = mergedWales, aes(fill = CymryCymraeg/N), lwd = 0) + coord_sf(crs = st_crs(mergedWales), datum = NA)+ scale_fill_gradientn(colours = rev(ocean.algae(20)), name = 'Cymry Cymraeg ', breaks = c(.05,.20,.35,.50)) + theme( text = element_text(size = 20, family = 'sans', colour = 'black'), panel.grid = element_blank(), rect = element_blank(), plot.background = element_rect(fill = "white")) -> MapCymryCymraeg

ggplot() + geom_sf(data = mergedWales, aes(fill = English/N), lwd = 0) + coord_sf(crs = st_crs(mergedWales), datum = NA)+ scale_fill_gradientn(colours = rev(ocean.dense(20)), name = 'English ', breaks = c(.05,.1,.15,.20)) + theme( text = element_text(size = 20, family = 'sans', colour = 'black'), panel.grid = element_blank(), rect = element_blank(), plot.background = element_rect(fill = "white")) -> MapEnglish

ggplot() + geom_sf(data = mergedWales, aes(fill = EthnicallyDiverse/N), lwd = 0) + coord_sf(crs = st_crs(mergedWales), datum = NA)+ scale_fill_gradientn(colours = rev(ocean.oxy(20)), name = 'Ethnically diverse ', breaks = c(.04,.08,.12,.16)) + theme( text = element_text(size = 20, family = 'sans', colour = 'black'), panel.grid = element_blank(), rect = element_blank(), plot.background = element_rect(fill = "white")) -> MapEthnicallyDiverse

tiff("FiveWalesMaps.tiff", width = 8, height = 8, units = 'in', res = 300, compression = 'lzw', type='cairo')

(MapAnglophoneWelsh | MapBritish) / ( MapCymryCymraeg | MapEnglish) / (MapEthnicallyDiverse|plot_spacer())

dev.off()

#Select just the data we want

dplyr::select(NSfW1719x, DvLSOA2011, CaseNo, NatIdWel, NatIdEng, NatIdBrit, Gender, WelSpk, GenHealth,

SampleAdultWeight.x, DvLA, EthnicityGrouped, Educat, DvMatDep,DvFinBilCred, IncResp,DvAgeGrp7, DvWIMDOvr5,

WelshFreq, LSOAName,Density, GenHealthD,LCA7, LCA5, MH, ThreeWalesRegion) -> NSfW1719xx

# Declare variables to include in imputation

Nominals <- c('Gender', 'Educat', 'DvMatDep', 'DvFinBilCred', 'GenHealthD', 'ThreeWalesRegion', 'LCA5','MH', 'EthnicityGrouped')

Ordinals <- c( 'IncResp', 'DvWIMDOvr5', 'DvAgeGrp7')

Numerics <- c('SampleAdultWeight.x', 'Density')

IDS <- c('CaseNo', 'DvLA', 'LSOAName', 'DvLSOA2011')

Variables <- c(Nominals, Ordinals, IDS, Numerics)

# Impute

impute.out <- amelia(NSfW1719xx[,Variables], idvars = IDS,noms =Nominals , ords = Ordinals, m = 5)

impute.out$imputations -> NSfW1719_imp

# Fit models to General health data

#Create lists to store model results

list() -> ModelsGH1

list() -> ModelsGH2

list() -> ModelsGH3

list() -> ModelsGH4

list() -> ModelsGH5

list() -> ModelsGH6

list() -> ModelsGH7

# Fit GH models to each imputation and store in lists

for(i in 1:length(NSfW1719_imp)){

print(i)

glmmTMB(GenHealthD ~ LCA5 + (1| DvLA/LSOAName), data= NSfW1719_imp[[i]], family = 'binomial', weights=SampleAdultWeight.x) -> ModelGH1N

glmmTMB(GenHealthD ~ LCA5 + DvAgeGrp7 + Gender + (1| DvLA/LSOAName), data= NSfW1719_imp[[i]], family = 'binomial', weights=SampleAdultWeight.x) -> ModelGH2N

glmmTMB(GenHealthD ~ LCA5 + DvAgeGrp7 + Gender + Educat + IncResp + (1| DvLA/LSOAName), data= NSfW1719_imp[[i]], family = 'binomial', weights=SampleAdultWeight.x) -> ModelGH3N

glmmTMB(GenHealthD ~ LCA5 + DvAgeGrp7 + Gender + Educat + IncResp + DvWIMDOvr5 + (1| DvLA/LSOAName), data= NSfW1719_imp[[i]], family = 'binomial', weights=SampleAdultWeight.x) -> ModelGH4N

glmmTMB(GenHealthD ~ LCA5 + DvAgeGrp7 + Gender + Educat + IncResp + DvWIMDOvr5 + scale(Density) + (1| DvLA/LSOAName), data= NSfW1719_imp[[i]], family = 'binomial', weights=SampleAdultWeight.x) -> ModelGH5N

glmmTMB(GenHealthD ~ LCA5 + DvAgeGrp7 + Gender + Educat + IncResp + DvWIMDOvr5 + scale(Density) + ThreeWalesRegion + (1| DvLA/LSOAName), data= NSfW1719_imp[[i]], family = 'binomial', weights=SampleAdultWeight.x) -> ModelGH6N

glmmTMB(GenHealthD ~ LCA5 + DvAgeGrp7 + Gender + Educat + IncResp + DvWIMDOvr5 + scale(Density) + ThreeWalesRegion + DvFinBilCred + DvMatDep + (1| DvLA/LSOAName), data= NSfW1719_imp[[i]], family = 'binomial', weights=SampleAdultWeight.x) -> ModelGH7N

ModelsGH1[[i]] <- ModelGH1N

ModelsGH2[[i]] <- ModelGH2N

ModelsGH3[[i]] <- ModelGH3N

ModelsGH4[[i]] <- ModelGH4N

ModelsGH5[[i]] <- ModelGH5N

ModelsGH6[[i]] <- ModelGH6N

ModelsGH7[[i]] <- ModelGH7N

}

# Make matrices to store estimates and SEs from each model

Estimates1 <- matrix(nrow = length(NSfW1719_imp), ncol = length(tidy(ModelsGH1[[i]])$term))

SEs1 <- matrix(nrow = length(NSfW1719_imp), ncol = length(tidy(ModelsGH1[[i]])$term))

Estimates2 <- matrix(nrow = length(NSfW1719_imp), ncol = length(tidy(ModelsGH2[[i]])$term))

SEs2 <- matrix(nrow = length(NSfW1719_imp), ncol = length(tidy(ModelsGH2[[i]])$term))

Estimates3 <- matrix(nrow = length(NSfW1719_imp), ncol = length(tidy(ModelsGH3[[i]])$term))

SEs3 <- matrix(nrow = length(NSfW1719_imp), ncol = length(tidy(ModelsGH3[[i]])$term))

Estimates4 <- matrix(nrow = length(NSfW1719_imp), ncol = length(tidy(ModelsGH4[[i]])$term))

SEs4 <- matrix(nrow = length(NSfW1719_imp), ncol = length(tidy(ModelsGH4[[i]])$term))

Estimates5 <- matrix(nrow = length(NSfW1719_imp), ncol = length(tidy(ModelsGH5[[i]])$term))

SEs5 <- matrix(nrow = length(NSfW1719_imp), ncol = length(tidy(ModelsGH5[[i]])$term))

Estimates6 <- matrix(nrow = length(NSfW1719_imp), ncol = length(tidy(ModelsGH6[[i]])$term))

SEs6 <- matrix(nrow = length(NSfW1719_imp), ncol = length(tidy(ModelsGH6[[i]])$term))

Estimates7 <- matrix(nrow = length(NSfW1719_imp), ncol = length(tidy(ModelsGH7[[i]])$term))

SEs7 <- matrix(nrow = length(NSfW1719_imp), ncol = length(tidy(ModelsGH7[[i]])$term))

#Extract estimates and SEs from lists and store them in matrices

for (i in 1:length(ModelsGH1)) {

tidy(ModelsGH1[[i]])$estimate -> Estimates1[i,]

tidy(ModelsGH1[[i]])$std.error -> SEs1[i,]

tidy(ModelsGH2[[i]])$estimate -> Estimates2[i,]

tidy(ModelsGH2[[i]])$std.error -> SEs2[i,]

tidy(ModelsGH3[[i]])$estimate -> Estimates3[i,]

tidy(ModelsGH3[[i]])$std.error -> SEs3[i,]

tidy(ModelsGH4[[i]])$estimate -> Estimates4[i,]

tidy(ModelsGH4[[i]])$std.error -> SEs4[i,]

tidy(ModelsGH5[[i]])$estimate -> Estimates5[i,]

tidy(ModelsGH5[[i]])$std.error -> SEs5[i,]

tidy(ModelsGH6[[i]])$estimate -> Estimates6[i,]

tidy(ModelsGH6[[i]])$std.error -> SEs6[i,]

tidy(ModelsGH7[[i]])$estimate -> Estimates7[i,]

tidy(ModelsGH7[[i]])$std.error -> SEs7[i,]

}

tidy(ModelsGH1[[i]])$term -> colnames(Estimates1)

tidy(ModelsGH2[[i]])$term -> colnames(Estimates2)

tidy(ModelsGH3[[i]])$term -> colnames(Estimates3)

tidy(ModelsGH4[[i]])$term -> colnames(Estimates4)

tidy(ModelsGH5[[i]])$term -> colnames(Estimates5)

tidy(ModelsGH6[[i]])$term -> colnames(Estimates6)

tidy(ModelsGH7[[i]])$term -> colnames(Estimates7)

#Pool estimates and SEs using Rubin's rule and convert to ORs with confidence intervals. Write out tabl for each model.

ModelGH1.ORs <- data.frame(CIlow=c(exp( mi.meld(q=Estimates1, se=SEs1, byrow=T)$q.mi - (mi.meld(q=Estimates1, se=SEs1, byrow=T)$se.mi*1.96))),

Estimate= c(exp( mi.meld(q=Estimates1, se=SEs1, byrow=T)$q.mi)),

CIhigh=c(exp(mi.meld(q=Estimates1, se=SEs1, byrow=T)$q.mi + (mi.meld(q=Estimates1, se=SEs1, byrow=T)$se.mi*1.96))), Terms = colnames(Estimates1))

write.table(ModelGH1.ORs, 'FiveWalesGH1.csv', sep = ',', row.names = F)

ModelGH2.ORs <- data.frame(CIlow=c(exp( mi.meld(q=Estimates2, se=SEs2, byrow=T)$q.mi - (mi.meld(q=Estimates2, se=SEs2, byrow=T)$se.mi*1.96))),

Estimate= c(exp( mi.meld(q=Estimates2, se=SEs2, byrow=T)$q.mi)),

CIhigh=c(exp(mi.meld(q=Estimates2, se=SEs2, byrow=T)$q.mi + (mi.meld(q=Estimates2, se=SEs2, byrow=T)$se.mi*1.96))), Terms = colnames(Estimates2))

write.table(ModelGH2.ORs, 'FiveWalesGH2.csv', sep = ',', row.names = F)

ModelGH3.ORs <- data.frame(CIlow=c(exp( mi.meld(q=Estimates3, se=SEs3, byrow=T)$q.mi - (mi.meld(q=Estimates3, se=SEs3, byrow=T)$se.mi*1.96))),

Estimate= c(exp( mi.meld(q=Estimates3, se=SEs3, byrow=T)$q.mi)),

CIhigh=c(exp(mi.meld(q=Estimates3, se=SEs3, byrow=T)$q.mi + (mi.meld(q=Estimates3, se=SEs3, byrow=T)$se.mi*1.96))), Terms = colnames(Estimates3))

write.table(ModelGH3.ORs, 'FiveWalesGH3.csv', sep = ',', row.names = F)

ModelGH4.ORs <- data.frame(CIlow=c(exp( mi.meld(q=Estimates4, se=SEs4, byrow=T)$q.mi - (mi.meld(q=Estimates4, se=SEs4, byrow=T)$se.mi*1.96))),

Estimate= c(exp( mi.meld(q=Estimates4, se=SEs4, byrow=T)$q.mi)),

CIhigh=c(exp(mi.meld(q=Estimates4, se=SEs4, byrow=T)$q.mi + (mi.meld(q=Estimates4, se=SEs4, byrow=T)$se.mi*1.96))), Terms = colnames(Estimates4))

write.table(ModelGH4.ORs, 'FiveWalesGH4.csv', sep = ',', row.names = F)

ModelGH5.ORs <- data.frame(CIlow=c(exp( mi.meld(q=Estimates5, se=SEs5, byrow=T)$q.mi - (mi.meld(q=Estimates5, se=SEs5, byrow=T)$se.mi*1.96))),

Estimate= c(exp( mi.meld(q=Estimates5, se=SEs5, byrow=T)$q.mi)),

CIhigh=c(exp(mi.meld(q=Estimates5, se=SEs5, byrow=T)$q.mi + (mi.meld(q=Estimates5, se=SEs5, byrow=T)$se.mi*1.96))), Terms = colnames(Estimates5))

write.table(ModelGH5.ORs, 'FiveWalesGH5.csv', sep = ',', row.names = F)

ModelGH6.ORs <- data.frame(CIlow=c(exp( mi.meld(q=Estimates6, se=SEs6, byrow=T)$q.mi - (mi.meld(q=Estimates6, se=SEs6, byrow=T)$se.mi*1.96))),

Estimate= c(exp( mi.meld(q=Estimates6, se=SEs6, byrow=T)$q.mi)),

CIhigh=c(exp(mi.meld(q=Estimates6, se=SEs6, byrow=T)$q.mi + (mi.meld(q=Estimates6, se=SEs6, byrow=T)$se.mi*1.96))), Terms = colnames(Estimates6))

write.table(ModelGH6.ORs, 'FiveWalesGH6.csv', sep = ',', row.names = F)

ModelGH7.ORs <- data.frame(CIlow=c(exp( mi.meld(q=Estimates7, se=SEs7, byrow=T)$q.mi - (mi.meld(q=Estimates7, se=SEs7, byrow=T)$se.mi*1.96))),

Estimate= c(exp( mi.meld(q=Estimates7, se=SEs7, byrow=T)$q.mi)),

CIhigh=c(exp(mi.meld(q=Estimates7, se=SEs7, byrow=T)$q.mi + (mi.meld(q=Estimates7, se=SEs7, byrow=T)$se.mi*1.96))), Terms = colnames(Estimates7))

write.table(ModelGH7.ORs, 'FiveWalesGH7.csv', sep = ',', row.names = F)

#Make figure 3

ModelGH1.ORs$Model <- '1'

ModelGH2.ORs$Model <- '2'

ModelGH3.ORs$Model <- '3'

ModelGH4.ORs$Model <- '4'

ModelGH5.ORs$Model <- '5'

ModelGH6.ORs$Model <- '6'

ModelGH7.ORs$Model <- '7'

rbind(ModelGH1.ORs, ModelGH2.ORs, ModelGH3.ORs, ModelGH4.ORs, ModelGH5.ORs, ModelGH6.ORs, ModelGH7.ORs) -> GHors

GHors[GHors$Terms %in% c('LCA5British', 'LCA5Cymry Cymraeg', 'LCA5English', 'LCA5Ethnically diverse'),] -> GHors

ggplot(aes(ymin =CIlow, ymax=CIhigh, y = Estimate, x = Model, colour = Model), data = GHors) + geom_pointrange() + scale_colour_manual(values = parula(7)) + geom_hline(yintercept=1, linetype='dashed') + facet_wrap(~Terms)

GHors$Terms <- factor(GHors$Terms, labels = c('British', 'Cymry Cymraeg', 'English', 'Ethnically diverse'))

tiff("FiveWales_ghORs.tiff", width = 12, height = 12, units = 'in', res = 300, compression = 'lzw', type='cairo')

ggplot(aes(ymin =CIlow, ymax=CIhigh, y = Estimate, x = Model, colour = Model), data = GHors) + geom_pointrange() + scale_colour_manual(values = parula(7)) + geom_hline(yintercept=1, linetype='dashed') + facet_wrap(~Terms) +theme(text=element_text(size = 22))

dev.off()

###################

#As above, but for mental health models

list() -> ModelsMH1

list() -> ModelsMH2

list() -> ModelsMH3

list() -> ModelsMH4

list() -> ModelsMH5

list() -> ModelsMH6

list() -> ModelsMH7

for(i in 1:length(NSfW1719_imp)){

print(i)

glmmTMB(MH ~ LCA5 + (1| DvLA/LSOAName), data= NSfW1719_imp[[i]], family = 'binomial', weights=SampleAdultWeight.x) -> ModelMH1N

glmmTMB(MH ~ LCA5 + DvAgeGrp7 + Gender + (1| DvLA/LSOAName), data= NSfW1719_imp[[i]], family = 'binomial', weights=SampleAdultWeight.x) -> ModelMH2N

glmmTMB(MH ~ LCA5 + DvAgeGrp7 + Gender + Educat + IncResp + (1| DvLA/LSOAName), data= NSfW1719_imp[[i]], family = 'binomial', weights=SampleAdultWeight.x) -> ModelMH3N

glmmTMB(MH ~ LCA5 + DvAgeGrp7 + Gender + Educat + IncResp + DvWIMDOvr5 + (1| DvLA/LSOAName), data= NSfW1719_imp[[i]], family = 'binomial', weights=SampleAdultWeight.x) -> ModelMH4N

glmmTMB(MH ~ LCA5 + DvAgeGrp7 + Gender + Educat + IncResp + DvWIMDOvr5 + scale(Density) + (1| DvLA/LSOAName), data= NSfW1719_imp[[i]], family = 'binomial', weights=SampleAdultWeight.x) -> ModelMH5N

glmmTMB(MH ~ LCA5 + DvAgeGrp7 + Gender + Educat + IncResp + DvWIMDOvr5 + scale(Density) + ThreeWalesRegion + (1| DvLA/LSOAName), data= NSfW1719_imp[[i]], family = 'binomial', weights=SampleAdultWeight.x) -> ModelMH6N

glmmTMB(MH ~ LCA5 + DvAgeGrp7 + Gender + Educat + IncResp + DvWIMDOvr5 + scale(Density) + ThreeWalesRegion + DvFinBilCred + DvMatDep + (1| DvLA/LSOAName), data= NSfW1719_imp[[i]], family = 'binomial', weights=SampleAdultWeight.x) -> ModelMH7N

ModelsMH1[[i]] <- ModelMH1N

ModelsMH2[[i]] <- ModelMH2N

ModelsMH3[[i]] <- ModelMH3N

ModelsMH4[[i]] <- ModelMH4N

ModelsMH5[[i]] <- ModelMH5N

ModelsMH6[[i]] <- ModelMH6N

ModelsMH7[[i]] <- ModelMH7N

}

Estimates1 <- matrix(nrow = length(NSfW1719_imp), ncol = length(tidy(ModelsMH1[[i]])$term))

SEs1 <- matrix(nrow = length(NSfW1719_imp), ncol = length(tidy(ModelsMH1[[i]])$term))

Estimates2 <- matrix(nrow = length(NSfW1719_imp), ncol = length(tidy(ModelsMH2[[i]])$term))

SEs2 <- matrix(nrow = length(NSfW1719_imp), ncol = length(tidy(ModelsMH2[[i]])$term))

Estimates3 <- matrix(nrow = length(NSfW1719_imp), ncol = length(tidy(ModelsMH3[[i]])$term))

SEs3 <- matrix(nrow = length(NSfW1719_imp), ncol = length(tidy(ModelsMH3[[i]])$term))

Estimates4 <- matrix(nrow = length(NSfW1719_imp), ncol = length(tidy(ModelsMH4[[i]])$term))

SEs4 <- matrix(nrow = length(NSfW1719_imp), ncol = length(tidy(ModelsMH4[[i]])$term))

Estimates5 <- matrix(nrow = length(NSfW1719_imp), ncol = length(tidy(ModelsMH5[[i]])$term))

SEs5 <- matrix(nrow = length(NSfW1719_imp), ncol = length(tidy(ModelsMH5[[i]])$term))

Estimates6 <- matrix(nrow = length(NSfW1719_imp), ncol = length(tidy(ModelsMH6[[i]])$term))

SEs6 <- matrix(nrow = length(NSfW1719_imp), ncol = length(tidy(ModelsMH6[[i]])$term))

Estimates7 <- matrix(nrow = length(NSfW1719_imp), ncol = length(tidy(ModelsMH7[[i]])$term))

SEs7 <- matrix(nrow = length(NSfW1719_imp), ncol = length(tidy(ModelsMH7[[i]])$term))

for (i in 1:length(ModelsMH1)) {

tidy(ModelsMH1[[i]])$estimate -> Estimates1[i,]

tidy(ModelsMH1[[i]])$std.error -> SEs1[i,]

tidy(ModelsMH2[[i]])$estimate -> Estimates2[i,]

tidy(ModelsMH2[[i]])$std.error -> SEs2[i,]

tidy(ModelsMH3[[i]])$estimate -> Estimates3[i,]

tidy(ModelsMH3[[i]])$std.error -> SEs3[i,]

tidy(ModelsMH4[[i]])$estimate -> Estimates4[i,]

tidy(ModelsMH4[[i]])$std.error -> SEs4[i,]

tidy(ModelsMH5[[i]])$estimate -> Estimates5[i,]

tidy(ModelsMH5[[i]])$std.error -> SEs5[i,]

tidy(ModelsMH6[[i]])$estimate -> Estimates6[i,]

tidy(ModelsMH6[[i]])$std.error -> SEs6[i,]

tidy(ModelsMH7[[i]])$estimate -> Estimates7[i,]

tidy(ModelsMH7[[i]])$std.error -> SEs7[i,]

}

tidy(ModelsMH1[[i]])$term -> colnames(Estimates1)

tidy(ModelsMH2[[i]])$term -> colnames(Estimates2)

tidy(ModelsMH3[[i]])$term -> colnames(Estimates3)

tidy(ModelsMH4[[i]])$term -> colnames(Estimates4)

tidy(ModelsMH5[[i]])$term -> colnames(Estimates5)

tidy(ModelsMH6[[i]])$term -> colnames(Estimates6)

tidy(ModelsMH7[[i]])$term -> colnames(Estimates7)

ModelMH1.ORs <- data.frame(CIlow=c(exp( mi.meld(q=Estimates1, se=SEs1, byrow=T)$q.mi - (mi.meld(q=Estimates1, se=SEs1, byrow=T)$se.mi*1.96))),

Estimate= c(exp( mi.meld(q=Estimates1, se=SEs1, byrow=T)$q.mi)),

CIhigh=c(exp(mi.meld(q=Estimates1, se=SEs1, byrow=T)$q.mi + (mi.meld(q=Estimates1, se=SEs1, byrow=T)$se.mi*1.96))), Terms = colnames(Estimates1))

write.table(ModelMH1.ORs, 'FiveWalesMH1.csv', sep = ',', row.names = F)

ModelMH2.ORs <- data.frame(CIlow=c(exp( mi.meld(q=Estimates2, se=SEs2, byrow=T)$q.mi - (mi.meld(q=Estimates2, se=SEs2, byrow=T)$se.mi*1.96))),

Estimate= c(exp( mi.meld(q=Estimates2, se=SEs2, byrow=T)$q.mi)),

CIhigh=c(exp(mi.meld(q=Estimates2, se=SEs2, byrow=T)$q.mi + (mi.meld(q=Estimates2, se=SEs2, byrow=T)$se.mi*1.96))), Terms = colnames(Estimates2))

write.table(ModelMH2.ORs, 'FiveWalesMH2.csv', sep = ',', row.names = F)

ModelMH3.ORs <- data.frame(CIlow=c(exp( mi.meld(q=Estimates3, se=SEs3, byrow=T)$q.mi - (mi.meld(q=Estimates3, se=SEs3, byrow=T)$se.mi*1.96))),

Estimate= c(exp( mi.meld(q=Estimates3, se=SEs3, byrow=T)$q.mi)),

CIhigh=c(exp(mi.meld(q=Estimates3, se=SEs3, byrow=T)$q.mi + (mi.meld(q=Estimates3, se=SEs3, byrow=T)$se.mi*1.96))), Terms = colnames(Estimates3))

write.table(ModelMH3.ORs, 'FiveWalesMH3.csv', sep = ',', row.names = F)

ModelMH4.ORs <- data.frame(CIlow=c(exp( mi.meld(q=Estimates4, se=SEs4, byrow=T)$q.mi - (mi.meld(q=Estimates4, se=SEs4, byrow=T)$se.mi*1.96))),

Estimate= c(exp( mi.meld(q=Estimates4, se=SEs4, byrow=T)$q.mi)),

CIhigh=c(exp(mi.meld(q=Estimates4, se=SEs4, byrow=T)$q.mi + (mi.meld(q=Estimates4, se=SEs4, byrow=T)$se.mi*1.96))), Terms = colnames(Estimates4))

write.table(ModelMH4.ORs, 'FiveWalesMH4.csv', sep = ',', row.names = F)

ModelMH5.ORs <- data.frame(CIlow=c(exp( mi.meld(q=Estimates5, se=SEs5, byrow=T)$q.mi - (mi.meld(q=Estimates5, se=SEs5, byrow=T)$se.mi*1.96))),

Estimate= c(exp( mi.meld(q=Estimates5, se=SEs5, byrow=T)$q.mi)),

CIhigh=c(exp(mi.meld(q=Estimates5, se=SEs5, byrow=T)$q.mi + (mi.meld(q=Estimates5, se=SEs5, byrow=T)$se.mi*1.96))), Terms = colnames(Estimates5))

write.table(ModelMH5.ORs, 'FiveWalesMH5.csv', sep = ',', row.names = F)

ModelMH6.ORs <- data.frame(CIlow=c(exp( mi.meld(q=Estimates6, se=SEs6, byrow=T)$q.mi - (mi.meld(q=Estimates6, se=SEs6, byrow=T)$se.mi*1.96))),

Estimate= c(exp( mi.meld(q=Estimates6, se=SEs6, byrow=T)$q.mi)),

CIhigh=c(exp(mi.meld(q=Estimates6, se=SEs6, byrow=T)$q.mi + (mi.meld(q=Estimates6, se=SEs6, byrow=T)$se.mi*1.96))), Terms = colnames(Estimates6))

write.table(ModelMH6.ORs, 'FiveWalesMH6.csv', sep = ',', row.names = F)

ModelMH7.ORs <- data.frame(CIlow=c(exp( mi.meld(q=Estimates7, se=SEs7, byrow=T)$q.mi - (mi.meld(q=Estimates7, se=SEs7, byrow=T)$se.mi*1.96))),

Estimate= c(exp( mi.meld(q=Estimates7, se=SEs7, byrow=T)$q.mi)),

CIhigh=c(exp(mi.meld(q=Estimates7, se=SEs7, byrow=T)$q.mi + (mi.meld(q=Estimates7, se=SEs7, byrow=T)$se.mi*1.96))), Terms = colnames(Estimates7))

write.table(ModelMH7.ORs, 'FiveWalesMH7.csv', sep = ',', row.names = F)

ModelMH1.ORs$Model <- '1'

ModelMH2.ORs$Model <- '2'

ModelMH3.ORs$Model <- '3'

ModelMH4.ORs$Model <- '4'

ModelMH5.ORs$Model <- '5'

ModelMH6.ORs$Model <- '6'

ModelMH7.ORs$Model <- '7'

rbind(ModelMH1.ORs, ModelMH2.ORs, ModelMH3.ORs, ModelMH4.ORs, ModelMH5.ORs, ModelMH6.ORs, ModelMH7.ORs) -> MHors

MHors[MHors$Terms %in% c('LCA5British', 'LCA5Cymry Cymraeg', 'LCA5English', 'LCA5Ethnically diverse'),] -> MHors

MHors$Terms <- factor(GHors$Terms, labels = c('British', 'Cymry Cymraeg', 'English', 'Ethnically diverse'))

tiff("FiveWales_MHors.tiff", width = 12, height = 12, units = 'in', res = 300, compression = 'lzw', type='cairo')

ggplot(aes(ymin =CIlow, ymax=CIhigh, y = Estimate, x = Model, colour = Model), data = MHors) + geom_pointrange() + scale_colour_manual(values = ocean.curl(8)) + geom_hline(yintercept=1, linetype='dashed') + facet_wrap(~Terms) +theme(text=element_text(size = 22))

dev.off()

########################

##########################

###Post-hoc analysis of Ethnically diverse group

summary(factor(NSfW1719_imp[[i]]$EthnicityGrouped[NSfW1719_imp[[1]]$LCA5=='Ethnically diverse']))

### General health

list() -> ModelsGH1ed

for(i in 1:length(NSfW1719_imp)){

print(i)

glmmTMB(GenHealthD ~ EthnicityGrouped + (1| DvLA/LSOAName), data= NSfW1719_imp[[i]][NSfW1719_imp[[1]]$LCA5=='Ethnically diverse',], family = 'binomial', weights=SampleAdultWeight.x) -> ModelGH1N

ModelsGH1ed[[i]] <- ModelGH1N

}

Estimates1 <- matrix(nrow = length(NSfW1719_imp), ncol = length(tidy(ModelsGH1ed[[i]])$term))

SEs1 <- matrix(nrow = length(NSfW1719_imp), ncol = length(tidy(ModelsGH1ed[[i]])$term))

for (i in 1:length(ModelsGH1ed)) {

tidy(ModelsGH1ed[[i]])$estimate -> Estimates1[i,]

tidy(ModelsGH1ed[[i]])$std.error -> SEs1[i,]

}

tidy(ModelsGH1ed[[i]])$term -> colnames(Estimates1)

ModelGH1.ORsed <- data.frame(CIlow=c(exp( mi.meld(q=Estimates1, se=SEs1, byrow=T)$q.mi - (mi.meld(q=Estimates1, se=SEs1, byrow=T)$se.mi*1.96))),

Estimate= c(exp( mi.meld(q=Estimates1, se=SEs1, byrow=T)$q.mi)),

CIhigh=c(exp(mi.meld(q=Estimates1, se=SEs1, byrow=T)$q.mi + (mi.meld(q=Estimates1, se=SEs1, byrow=T)$se.mi*1.96))), Terms = colnames(Estimates1))

###################

###Mental health

list() -> ModelsMH1ed

for(i in 1:length(NSfW1719_imp)){

print(i)

glmmTMB(MH ~ EthnicityGrouped + (1| DvLA/LSOAName), data= NSfW1719_imp[[i]][NSfW1719_imp[[1]]$LCA5=='Ethnically diverse',], family = 'binomial', weights=SampleAdultWeight.x) -> ModelMH1N

ModelsMH1ed[[i]] <- ModelMH1N

}

Estimates1 <- matrix(nrow = length(NSfW1719_imp), ncol = length(tidy(ModelsMH1ed[[i]])$term))

SEs1 <- matrix(nrow = length(NSfW1719_imp), ncol = length(tidy(ModelsMH1ed[[i]])$term))

for (i in 1:length(ModelsMH1ed)) {

tidy(ModelsMH1ed[[i]])$estimate -> Estimates1[i,]

tidy(ModelsMH1ed[[i]])$std.error -> SEs1[i,]

}

tidy(ModelsMH1ed[[i]])$term -> colnames(Estimates1)

ModelMH1.ORsed <- data.frame(CIlow=c(exp( mi.meld(q=Estimates1, se=SEs1, byrow=T)$q.mi - (mi.meld(q=Estimates1, se=SEs1, byrow=T)$se.mi*1.96))),

Estimate= c(exp( mi.meld(q=Estimates1, se=SEs1, byrow=T)$q.mi)),

CIhigh=c(exp(mi.meld(q=Estimates1, se=SEs1, byrow=T)$q.mi + (mi.meld(q=Estimates1, se=SEs1, byrow=T)$se.mi*1.96))), Terms = colnames(Estimates1))
